# Mining for gene-environment and gene-gene interactions: parametric and non-parametric tests for detecting variance quantitative trait loci

**DOI:** 10.3389/fgene.2025.1617504

**Published:** 2025-09-01

**Authors:** Wan-Yu Lin

**Affiliations:** ^1^ Institute of Health Data Analytics and Statistics, College of Public Health, National Taiwan University, Taipei, Taiwan; ^2^ Master of Public Health Program, College of Public Health, National Taiwan University, Taipei, Taiwan

**Keywords:** cholesterol, dyslipidemia, triglyceride, variance quantitative trait locus, Taiwan Biobank

## Abstract

**Introduction:**

Detection of variance quantitative trait loci (vQTL) can facilitate the discovery of gene-environment (GxE) and gene-gene interactions (GxG). Identifying vQTLs before direct GxE and GxG analyses can considerably reduce the number of tests and the multiple-testing penalty.

**Methods:**

Despite some methods proposed for vQTL detection, few studies have performed a head-to-head comparison simultaneously concerning false positive rates (FPRs), power, and computational time. This work compares three parametric and two non-parametric vQTL tests.

**Results:**

Simulation studies show that the deviation regression model (DRM) and Kruskal-Wallis test (KW) are the most recommended parametric and non-parametric tests, respectively. The quantile integral linear model (QUAIL, non-parametric) appropriately preserves the FPR under normally or non-normally distributed traits. However, its power is never among the optimal choices, and its computational time is much longer than that of competitors. The Brown-Forsythe test (BF, parametric) can suffer from severe inflation in FPR when SNP’s minor allele frequencies <0.2. The double generalized linear model (DGLM, parametric) is not valid for non-normally distributed traits, although it is the most powerful method for normally distributed traits.

**Discussion:**

Considering the robustness (to outliers) and computation time, I chose KW to analyze four lipid traits in the Taiwan Biobank. I further showed that GxE and GxG were enriched among 30 vQTLs identified from the four lipid traits.

## 1 Introduction

Omitting essential predictors from a regression model can lead to heteroscedasticity (Breusch and Pagan, 1979). In genetic analyses, loci with unequal phenotypic variances across different genotype groups are called “variance quantitative trait loci” (vQTLs), which can be caused by omitting gene-environment interaction (GxE) or gene-gene interaction (GxG) in the regression model (Pare et al., 2010; Ronnegard and Valdar, 2012; Shi, 2022; Wang et al., 2019). For example, Wang et al. performed a genome-wide vQTL analysis of 5.6 million variants on ∼350,000 unrelated individuals of European ancestry for 13 quantitative traits. They identified 75 significant vQTLs for nine traits, especially those related to obesity. Pervasive GxE effects for obesity-related traits were further explored through direct GxE analyses. Wang et al.‘s study has demonstrated the detection of GxE without environmental data (Wang et al., 2019).

Methods for detecting vQTLs are categorized as parametric or non-parametric. The development of parametric vQTL tests usually depends on the assumption of normally distributed traits (Marderstein et al., 2021; Young et al., 2018). Contrastingly, non-parametric vQTL tests are generally more robust to trait distributions (Miao et al., 2022).

For every single-nucleotide polymorphism (SNP), we can use the Brown-Forsythe (BF) test (Brown and Forsythe, 1974) to compute the dispersion within three genotype groups. Compared with the Levene’s test (Levene, 1960), the BF test is more robust to outliers by choosing the median to replace the mean as the center of each genotype group. Moreover, a “deviation regression model” (DRM) has been proposed to allow continuous predictors such as minor allele dosages (Marderstein et al., 2021). Therefore, the predictor is not limited to minor allele counts of SNPs (i.e., 0, 1, and 2). Furthermore, a double generalized linear model (DGLM) was also developed to identify loci associated with trait variability and to detect interactions in genome-wide association studies (GWAS) (Ronnegard and Valdar, 2011; Smyth, 1989). BF, DRM, and DGLM are all the so-called parametric vQTL methods.

Non-parametric methods for vQTL identification include the Kruskal-Wallis test (KW) (Kruskal and Wallis, 1952) and a quantile integral linear model (QUAIL) (Miao et al., 2022). As the non-parametric counterpart of one-way analysis of variance (ANOVA), KW is used to compare the median difference between multiple groups. As long as we first calculate the deviation between trait values and within-group medians, KW can also be applied to test phenotypic homoscedasticity among the three genotype groups. Another non-parametric method, QUAIL, assesses genetic effects on phenotypic variability based on the quantile regression framework. It allows covariate adjustment, non-normally distributed traits, and continuous predictors (i.e., not limited to three genotype categories) (Miao et al., 2022). However, to integrate information from *K* quantiles (usually *K* = 100), QUAIL’s computation time is much longer than that of its competitors (BF, DRM, DGLM, and KW). Implementing QUAIL for genome-wide vQTL analysis is computationally challenging.

This work evaluates the performance of the abovementioned parametric or non-parametric methods in detecting vQTLs through Monte Carlo simulations. I performed a head-to-head comparison to assess the false positive rates (FPRs), power, and computation time across several popular vQTL methods. Furthermore, I apply these approaches to four lipid traits in the Taiwan Biobank (TWB) data, including high-density lipoprotein cholesterol (HDL), low-density lipoprotein cholesterol (LDL), triglyceride (TG), and total cholesterol (TCHO). After identifying vQTLs for these four traits, I perform direct GxE and GxG analyses to demonstrate enriched interaction effects among vQTLs.

## 2 Materials and methods

### 2.1 Covariate-adjusted residuals

Let $Y_{j}$ be the continuous trait of the *j*th individual, $G_{1,j}$ and $G_{2,j}$ be two indicator variables categorizing three genotype groups of a SNP, and $X_{j}$ be the *C*-length covariate vector of individual *j* (assuming *C* covariates should be adjusted). $G_{1,j}$ and $G_{2,j}$ can be coded as (0, 0), (1, 0), and (0, 1) for 0, 1, and 2 copies of a SNP’s specific allele. According to previous studies, the FPR of detecting vQTLs may be inflated in the presence of a large SNP’s main effect (Ronnegard and Valdar, 2011). Therefore, I remove SNP’s main effect and covariates’ effects from $Y_{j}$ to ensure that the identification of vQTLs will not be confounded by other factors. Specifically, I extract the residuals of regressing $Y_{j}$ on $G_{1,j}$ , $G_{2,j}$ , and $X_{j}$ . With this preliminary procedure, all parametric or non-parametric vQTL tests have the potential to adjust for covariates such as age, sex, and ancestry principal components (PCs).

### 2.2 Non-parametric vQTL methods

Non-parametric vQTL methods are developed without the assumption of normally distributed traits. Therefore, they are generally more robust to trait distributions (Miao et al., 2022). Like many non-parametric statistical tests, they may be less powerful than parametric vQTL methods when the traits indeed follow normal distributions (Ronnegard and Valdar, 2012). However, they can have more valid results even when the traits are not normally distributed. In the following, I introduce two non-parametric methods, including the Kruskal-Wallis test (KW) and the quantile integral linear model (QUAIL).

#### 2.2.1 Kruskal-Wallis test (KW)

KW is a non-parametric statistical test used to assess whether there is a significant difference between the medians of two or more independent groups. Because the KW test is not originally designed to detect differences in variances, I have to convert the data into a measure of “dispersion” before using KW. Let $e_{\left(i\right)j}$ be the “covariate-adjusted residual” (obtained from Section 2.1) of individual *j* with genotype *i*, $\tilde{e_{i}}$ be the median of “covariate-adjusted residuals” within genotype group *i*. The deviation between individual *j*’s covariate-adjusted residual and his/her group median is $D_{\left(i\right)j}=\left|e_{\left(i\right)j}-\tilde{e_{i}}\right|$ (a measure of dispersion), where j=1,⋯,N and usually i=1,2,3 (subscript *j* is sufficient to distinguish all *N* individuals; subscript *i* is supplementary to indicate the genotype group). Suppose $r_{\left(i\right)j}$ is the rank of $D_{\left(i\right)j}$ counting from all individuals across three genotype groups. The KW test statistic (Kruskal and Wallis, 1952) is listed in Equation 1 as follows,

$$\text{KW}=\left(N-1\right)\frac{\sum_{i=1}^{M}n_{i}{\left(\bar{r_{i.}}-\bar{r}\right)}^{2}}{\sum_{i=1}^{M}\sum_{j=1}^{n_{i}}{\left(r_{\left(i\right)j}-\bar{r}\right)}^{2}}$$ (1)

where *N* is the total sample size, $n_{i}$ is the number of individuals in genotype group *i*, *M* is the number of genotype groups (usually *M* = 3), $\bar{r_{i.}}=\sum_{j=1}^{n_{i}}r_{\left(i\right)j}/n_{i}$ is the average of ranks in genotype group *i*, $\bar{r}=\sum_{i=1}^{M}\sum_{j=1}^{n_{i}}r_{\left(i\right)j}/N=\frac{1}{2}\left(N+1\right)$ is the average of ranks among all individuals. The KW test statistic (1) follows the chi-square distribution with the degrees of freedom $\left(M-1\right)$ . All analyses in this work were conducted using R (version 4.3.1). The KW test was implemented with the R function “kruskal.test” in the R environment.

#### 2.2.2 Quantile integral linear model (QUAIL)

Although I have adjusted the covariates’ effects in Section 2.1, I still present the statistical model shown in QUAIL’s paper as follows (Miao et al., 2022). Through this model, people may get a complete understanding of Miao et al.’s methodology. Let the conditional quantile function (*Q*) of the trait *Y* at a SNP be Equation 2 as follows

$$Q_{Y}\left(\tau |genotype=i,X\right)=\mu_{\tau }+i\beta_{\tau }+X\alpha_{\tau }$$ (2)

where τ is the quantile ranging from 0 to 0.5, i=1,2,3 , $\mu_{\tau }$ is the intercept, $\beta_{\tau }$ is the coefficient of the quantile regression, ***X*** is the N×C matrix for *C* covariates, and $\alpha_{\tau }$ is the *C*-length vector for covariate effects. QUAIL measures $\left(\beta_{1-\tau }-\beta_{\tau }\right)$ given $\tau \in \left(0,0.5\right)$ , i.e., the difference between the regression coefficients of the $\left(1-\tau \right)$ ^th^ and τ ^th^ quantiles. QUAIL tests the hypothesis $H_{0}:\beta_{QI}=0$ vs $H_{1}:\beta_{QI}\neq 0$ , where $\beta_{QI}$ in Equation 3 is the quantile-integrated effect, i.e.,

$$\beta_{QI}=\int_{0}^{0.5}\left(\beta_{1-\tau }-\beta_{\tau }\right)d\tau$$ (3)

$\beta_{QI}$ is the integral of $\left(\beta_{1-\tau }-\beta_{\tau }\right)$ from τ=0 to τ=0.5 , which can be estimated by summing the areas of *K* rectangles with a length of $\left(\hat{\beta_{1-\tau_{k}}}-\hat{\beta_{\tau_{k}}}\right)$ (where *k* = 1, … , *K*) and a width of $\left(0.5-0\right)/K=1/2K$ . That is, $\beta_{QI}$ can be estimated through $\hat{\beta_{QI}}=\sum_{k=1}^{K}\left(\hat{\beta_{1-\tau_{k}}}-\hat{\beta_{\tau_{k}}}\right)/2K$ . To have a more accurate estimation for $\beta_{QI}$ , *K* is required to be a large number. In the QUAIL R code, Miao et al. used *K* = 100 as the default setting (Miao et al., 2022). The QUAIL R code was downloaded from GitHub (https://github.com/qlu-lab/QUAIL).

### 2.3 Parametric vQTL methods

Parametric vQTL methods are usually developed with the assumption of normally distributed traits (Marderstein et al., 2021; Young et al., 2018). Therefore, their performances are generally more sensitive to trait distributions (Miao et al., 2022). However, if the trait really follows the normal distribution, parametric tests can be more powerful than their non-parametric counterparts. In the following, I describe three parametric vQTL methods, including the deviation regression model (DRM), Brown-Forsythe test (BF), and the double generalized linear model (DGLM).

#### 2.3.1 Deviation regression model (DRM)

To search for SNPs that are associated with phenotypic variability, Marderstein et al. regressed $D_{\left(i\right)j}$ on the minor allele count at each SNP (i.e., 0, 1, or 2) (Marderstein et al., 2021). The regression model is $D_{\left(i\right)j}=\alpha +\beta \cdot \left(i-1\right)+\varepsilon_{j}$ , where the predictor $\left(i-1\right)$ is the minor allele count (i.e., 0, 1, or 2) and the random error term $\varepsilon_{j}$ follows a normal distribution with a mean of 0 and a variance of $\sigma_{\varepsilon }^{2}$ . Because $D_{\left(i\right)j}$ is the deviation between individuals’ covariate-adjusted residuals and their group medians, this approach is called the deviation regression model (DRM). DRM tests whether the regression coefficient β is statistically significant, i.e., $H_{0}:\beta =0$ vs $H_{0}:\beta \neq 0$ . The DRM R code was downloaded from GitHub (https://github.com/drewmard/DRM).

#### 2.3.2 Brown-Forsythe test (BF)

The BF test statistic is listed in Equation 4 as follows,

$$\text{BF}=\frac{\sum_{i=1}^{M}n_{i}{\left(\bar{D_{i.}}-\bar{D}\right)}^{2}/\left(M-1\right)}{\sum_{i=1}^{M}\sum_{j=1}^{n_{i}}{\left(D_{\left(i\right)j}-\bar{D_{i.}}\right)}^{2}/\left(N-M\right)}$$ (4)

where $\bar{D_{i.}}=\sum_{j=1}^{n_{i}}D_{\left(i\right)j}/n_{i}$ is the average of deviations in genotype group *i*, $\bar{D}=\sum_{i=1}^{M}\sum_{j=1}^{n_{i}}D_{\left(i\right)j}/N$ is the average of deviations in all individuals. The BF test statistic (4) follows the *F* distribution with the numerator degrees of freedom $\left(M-1\right)$ , and the denominator degrees of freedom $\left(N-M\right)$ (Brown and Forsythe, 1974). The BF test is equivalent to the ANOVA *F* test for regressing $D_{ij}$ on two indicator variables that separate the three genotype groups of an SNP. Therefore, BF treats genotypes as a categorical scale (by using two indicator variables), while DRM regards genotypes as a continuous scale (by coding genotypes as 0, 1, or 2).

To implement the BF test, I used the R function “leveneTest” while specifying “center = median” in the “car” package (version 3.1–3) (Fox and Weisberg, 2018). The Levene’s and the BF tests are both used to test the homoscedasticity across different groups. The BF test is more robust to outliers than Levene’s test because it uses deviations from the group median, while Levene’s test uses deviations from the group mean.

#### 2.3.3 Double generalized linear model (DGLM)

DGLM fits two generalized linear models (GLM) that model genetic effects on the mean and dispersion of the covariate-adjusted residuals (Zhang and Bell, 2024). It regresses the covariate-adjusted residual $e_{\left(i\right)j}$ on the minor allele count at each SNP (i.e., 0, 1, or 2) while allowing the dispersion of $e_{\left(i\right)j}$ varies with the minor allele count. The regression model is $e_{\left(i\right)j}=\alpha +\beta \cdot \left(i-1\right)+\varepsilon_{j}$ , where the random error term $\varepsilon_{j}$ follows a normal distribution with a mean of 0 and a variance of $\sigma_{i}^{2}$ (allowing each genotype group has its variance). The DGLM tests whether the variance of $e_{\left(i\right)j}$ varies with the predictor (i.e., the minor allele count at each SNP). The DGLM method was implemented with the “*dglm*” R package (version: 1.8.6).

### 2.4 Data from the Taiwan Biobank (TWB)

As of February 2024, 147,836 individuals aged *30 to 70 years* have been genotyped whole-genome. The TWB performed genotype imputation with the IMPUTE2 software (v2.3.1) (Delaneau et al., 2013; Howie et al., 2009). To improve the imputation accuracy, the TWB combined 1,445 TWB individuals with whole-genome sequence data and 504 East Asians (EAS) from the 1,000 Genomes Phase 3 v5 as the reference panel (Wei et al., 2021). After completing the imputation, the TWB excluded variants with missing rates >5%, minor allele frequencies (MAFs) < 0.01%, or imputation information scores <0.3, where 0.3 was usually adopted as the acceptable threshold for imputation quality (Kosugi et al., 2023). After this quality control filtering, we had 9,814,944 autosomal variants for analysis.

### 2.5 Simulation studies

I performed simulations to evaluate the type I error rates and power of the five abovementioned methods. I here focused on the simulations of detecting SNP1-by-SNP2 interactions. Nonetheless, the results can be generalized to GxE identification. I randomly selected four SNPs as SNP1, including rs34625133 on chromosome (chr.) 1, rs7870809 on chr. 9, rs7982209 on chr. 13, and rs140100 on chr. 22. The MAFs of these four SNPs were 0.1, 0.2, 0.3, and 0.4, respectively. They were, in turn (one by one), treated as SNP1. I aimed to evaluate the power of detecting SNP1-by-SNP2 interactions under various MAFs of SNP1.

I used 20,000 common SNPs (MAFs ≥ 0.05) on chr. 1 as SNP2 and generated traits as Equation 5,

$$Y_{j}=\beta_{1}{SNP}_{1,j}+\beta_{2}{SNP}_{2,j}+\beta_{\mathrm{INT}}{SNP}_{1,j}\times {SNP}_{2,j}+\varepsilon_{j},j=1,\cdots ,30000\;\left(\text{or }147836\right)$$ (5)

where ${SNP}_{1,j}=0$ , 1, or 2 represented individual *j*’s minor allele count at SNP1, and ${SNP}_{2,j}=0$ , 1, or 2 was his/her minor allele count at SNP2. I considered two levels of sample size: *N* = 30,000 or 147,836, in which 147,836 was the number of TWB individuals with genotyping data. The coefficients $\beta_{1}$ and $\beta_{2}$ are main effects of SNP1 and SNP2, respectively. I investigated two situations: (1) with SNP main effects ( $\beta_{1}=\beta_{2}=0.3$ ); and (2) without SNP main effects ( $\beta_{1}=\beta_{2}=0$ ). Given an individual’s genotypes, $\beta_{1}{SNP}_{1,j}+\beta_{2}{SNP}_{2,j}+\beta_{\mathrm{INT}}{SNP}_{1,j}\times {SNP}_{2,j}$ is the deterministic part and $\varepsilon_{j}$ is the stochastic part of the trait. Following Miao et al. (Miao et al., 2022), I considered three settings for the random error term $\varepsilon_{j}$ : (1) a standard normal distribution; (2) a *t* distribution with the degrees of freedom 3 (a kurtotic distribution); (3) a chi-square distribution with the degrees of freedom 6 (a skewed distribution). Because $\varepsilon_{j}$ is the only stochastic part of the trait, the distribution of the trait is determined by the distribution of $\varepsilon_{j}$ . A kurtotic distribution such as the *t* distribution has heavier tails than the normal distribution. This phenomenon mimics the fact that more extreme values exist than the normal distribution. The chi-square distribution is right-skewed like the pattern of many phenotypes, such as cholesterol (Tharu and Tsokos, 2017) and body weight (Kozlowski and Gawelczyk, 2002). To fix the phenotypic variance explained by SNPs across different distributions of $\varepsilon_{j}$ , I followed Miao et al. (Miao et al., 2022) to perform the *z*-score transformation on $\varepsilon_{j}$ .

By specifying $\beta_{\mathrm{INT}}=0$ , I plotted the QQ plot to examine the p-values under the null hypothesis. On the other hand, $\beta_{\mathrm{INT}}=0.3$ was assumed when evaluating the power of the vQTLs methods. To sum up, 12 scenarios (two levels of sample size × two situations of main effects × three kinds of trait distributions) were simulated to evaluate the performance of the five vQTL methods. Each scenario was simulated 20,000 times. The percentage of the variance explained by the interaction effect was approximately 0.3%, 0.6%, 0.9%, and 1.2% when the MAF of SNP1 was 0.1, 0.2, 0.3, and 0.4, respectively. The coefficients in Equation (5) (i.e., $\beta_{1}$ , $\beta_{2}$ , and $\beta_{\mathrm{INT}}$ ) were used to generate phenotype *Y*. Instead of testing any coefficient, in simulations, I tested whether phenotypic variance varied with genotype groups.

Although Equation (5) is originally designed to test for GxG interactions, the results can be generalized to GxE identification. Without loss of generality, I can replace ${SNP}_{2}$ in Equation (5) with an environmental factor having three possible levels: 0, 1, and 2. Therefore, the simulation results can be generalized to GxE identification.

## 3 Results

### 3.1 Simulation studies

#### 3.1.1 False positive rates

**FIGURE 1:**
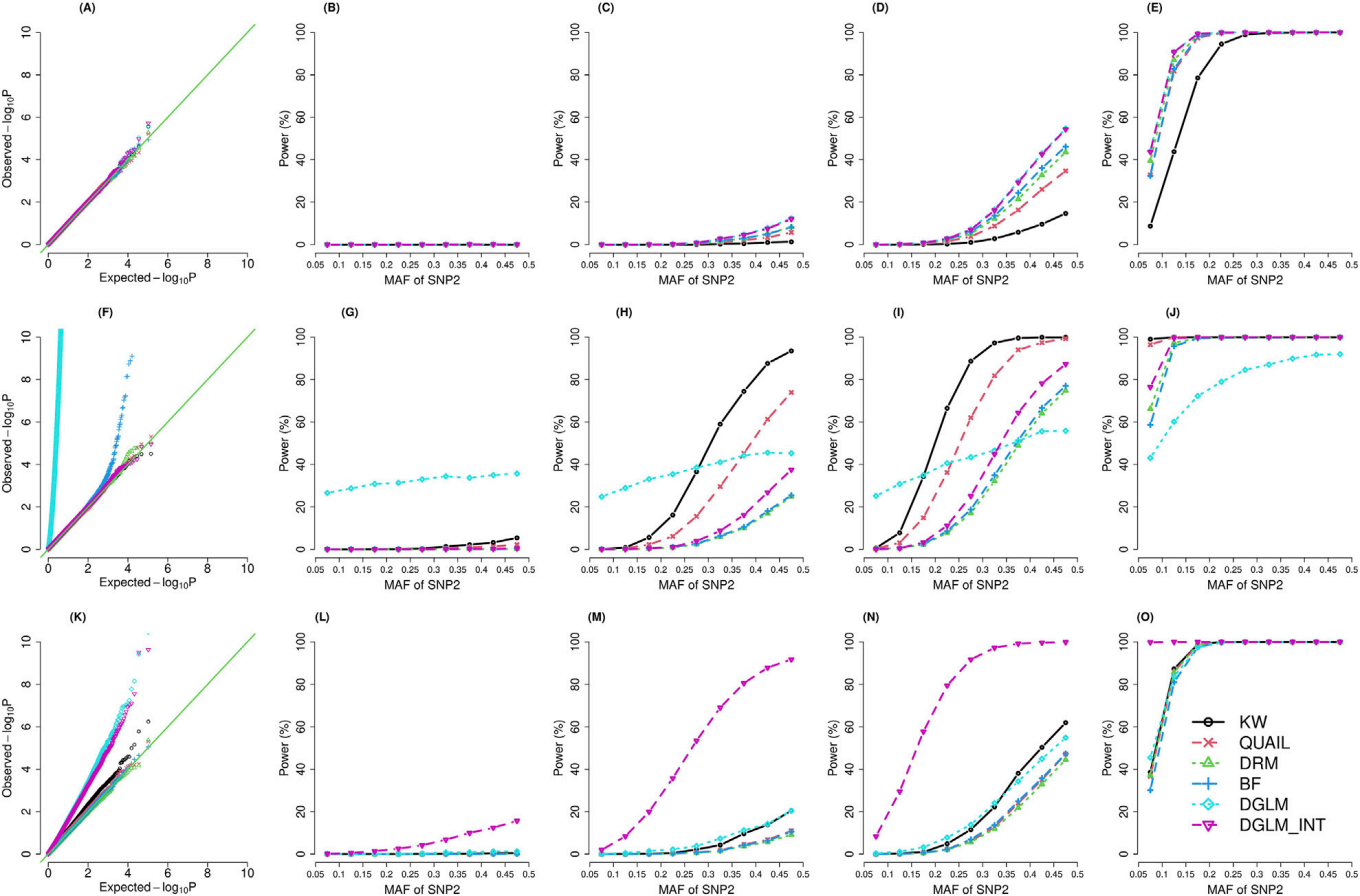
QQ plots (the left column) and power ( α = 5E-8; columns 2–5) for vQTL tests (*N* = 30,000; with SNP main effects). The distribution for the error term: (top row) a standard normal distribution; (middle row) a *t* distribution with the degrees of freedom 3; (bottom row) a chi-square distribution with the degrees of freedom 6. (A) Normal dist., QQ plot. (B) Normal dist., MAF of SNP1 = 0.1. (C) Normal dist., MAF of SNP1 = 0.2. (D) Normal dist., MAF of SNP1 = 0.3. (E) Normal dist., MAF of SNP1 = 0.4. (F) t dist., QQ plot. (G) t dist., MAF o SNP1 = 0.1. (H) t dist., MAF o SNP1 = 0.2. (I) t dist., MAF o SNP1 = 0.3. (J) t dist., MAF o SNP1 = 0.4. (K) chi-square dist., QQ plot. (L) chi-square dist., MAF of SNP1 = 0.1. (M) chi-square dist., MAF of SNP1 = 0.2. (N) chi-square dist., MAF of SNP1 = 0.3. (O) chi-square dist., MAF of SNP1 = 0.4.

Figure 1 presents the QQ plots (the left column) and power under α = 5E-8 (columns 2–5) for five vQTL tests (*N* = 30,000; with SNP main effects). Supplementary Figure S1 demonstrates the results given *N* = 30,000, and SNP main effects do not exist. Supplementary Figure S2 (with SNP main effects) and S3 (without SNP main effects) show the results when *N* = 147,836. The rank-based inverse-normal transformation (INT) can convert data to a normal distribution. However, previous vQTL studies found that this INT transformation led to inflation in false positive rates (FPR) (Marderstein et al., 2021; Miao et al., 2022; Wang et al., 2019). The FPR was calculated by dividing the incorrectly classified negatives by the total negatives. To evaluate INT’s performance in our simulation setting, we equipped it with DGLM.

##### 3.1.1.1 Parametric tests

The results show that DGLM has inflated FPR when the phenotypes are not normally distributed (Figure 1; Supplementary Figures S1–S3F, K). Taking the INT transformation helps to adjust FPR for kurtotic phenotypes (Figure 1; Supplementary Figures S1–S3F) but not for skewed phenotypes (Figure 1; Supplementary Figures S1–S3K).

The BF test has inflated FPR given kurtotic phenotypes (Figure 1; Supplementary Figures S1–S3F), and the situation is getting worse for smaller sample sizes (Figure 1; Supplementary Figure S1F). Supplementary Figures S4–S15 demonstrate the QQ plots stratified by the MAF range. For the BF test, the inflation in FPR is especially severe for kurtotic phenotypes when *N* = 30,000 and MAF <0.2 (Supplementary Figures S5, S8, (A) (B) (C)). Given *N* = 30,000 and a small MAF, a genotype group may only contain a few observations, and false positives may come with the issue of data sparsity (Lin, 2024).

DRM is similar to BF, except that DRM treats genotypes as a continuous scale (0, 1, or 2). DRM does not categorize the three genotypes into three groups. Therefore, the sparsity within genotype groups is not a critical problem for DRM, and the inflation in FPR is not critical for DRM compared with BF (Supplementary Figures S5, S8A–C).

##### 3.1.1.2 Non-parametric tests

QUAIL preserves an appropriate FPR across three trait distributions. KW maintains a suitable FPR under the kurtotic distribution (Figure 1; Supplementary Figure S1–S3F) but has a slight inflation in FPR given the skewed distribution (Figure 1; Supplementary Figure S1–S3K). Under a kurtotic distribution, more extreme values can occur compared to the normal distribution. Nonetheless, by transforming the extreme values into ranks, KW can still preserve an appropriate FPR. By contrast, previous research has found that KW tends to have inflated FPR for heteroscedastic cases (McDonald, 2014). Under a skewed distribution, unequal variances in ranks across the three genotype groups may still exist even if there is no GxG or GxE. KW’s strategy to transform values into ranks cannot entirely address the heteroscedastic problem.

#### 3.1.2 Statistical power

The statistical power was calculated by dividing the correctly classified positives by the total positives (each scenario was simulated 20,000 times). When the phenotype is normally distributed, DGLM and DGLM_INT were the most powerful methods (Figure 1; Supplementary Figure S1–S3C–E). Among the two non-parametric tests, QUAIL was superior to KW for normally distributed phenotypes (Figure 1; Supplementary Figure S1–S3C–E). When the phenotype is a kurtotic distribution, DGLM and BF had inflation in FPR (Figure 1; Supplementary Figure S1–S3F). KW became the most outstanding test among the four valid methods that preserve a suitable FPR (Figure 1; Supplementary Figure S1–S3F–J). When the phenotype is skewed, KW had a slight inflation in FPR, whereas DGLM and DGLM_INT had a severe inflation in FPR (Figure 1; Supplementary Figure S1–S3K). The other three valid methods (QUAIL, DRM, and BF) had similar power (Figure 1; Supplementary Figure S1–S3L–O).

By comparing the results of different simulation scenarios, I concluded three points: (1) When the MAF for SNP1 or SNP2 was larger, the percentage of the variance explained by the interaction effect increased, and the power of each method was enhanced. (2) When the sample size was enlarged from *N* = 30,000 to *N* = 147,836, the power performances of all methods were greatly improved. (3) Lastly, by comparing Figure 1 with Supplementary Figure S1, S2 with Supplementary Figure S3, the power of each method was higher in the presence of SNP main effects than in the absence of SNP main effects.

#### 3.1.3 Computational time

Table 1 shows the computation time (in seconds) for each simulation replicate. I measured the execution time in R (version 4.3.1) on a Linux system running at 3.6 GHz and 32 GB of RAM. Although QUAIL is a valid test with appropriate FPR under three trait distributions, it takes much more computation time than other methods. The other non-parametric test, KW, requires only 1/70–1/90 of QUAIL’s execution time. The computation time of the four parametric tests is also reasonable. The time of computation needed for these tests is ordered as DRM < BF < KW < DGLM ≈ DGLM_INT <<<<< QUAIL. Table 2 summarizes the performance of each method in the simulation on FPR, power, and computational time under different scenarios.

**TABLE 1:** Computation time (in seconds) for each simulation replicate The execution time was measured in R (version 4.3.1) on a Linux system running at 3.6 GHz and 32 GB of RAM.

Phenotype distribution	*N*	KW	QUAIL	DRM	BF	DGLM	DGLM_INT
Normal	30,000	0.076	5.372	0.018	0.020	0.151	0.162
T	30,000	0.092	8.252	0.024	0.028	0.273	0.214
Chi-square	30,000	0.075	5.485	0.018	0.021	0.163	0.168
Normal	147,836	0.402	33.464	0.096	0.105	0.824	0.891
T	147,836	0.399	33.950	0.094	0.103	1.090	0.875
Chi-square	147,836	0.411	35.913	0.098	0.106	0.884	0.929

**TABLE 2:** Summary of the simulation results.

	KW	QUAIL	DRM	BF	DGLM	DGLM_INT
False positive rate (FPR)	Slight inflation in FPR given skewed distributions	Valid	Inflation in FPR is not critical for DRM compared with BF.	Inflation in FPR is especially severe for kurtotic traits when N = 30,000 and MAF <0.2	Valid only when the trait is normally distributed	Severe inflation in FPR given skewed distributions
Statistical power	Optimal for kurtotic distributions	Never among the optimal choices	Similar to BF.	Similar to DRM.	Optimal for normally distributed traits	Optimal for normally distributed traits
Computational time (ranking from shortest to longest)	3	6 (longest)	1 (shortest)	2	4	4

### 3.2 Real data analysis

#### 3.2.1 30 vQTLs of four lipid traits

When conducting actual genome-wide data analysis, investigators had to exclude cryptic relatedness among individuals (Marees et al., 2018). The TWB investigated cryptic relatedness among participants with the KING (Kinship-based INference for GWAS) software (Manichaikul et al., 2010). I removed the person with higher missing genotype rates for each first- or second-degree relative pair. This step excluded 28,928 from the 147,836 TWB individuals, and 118,908 remained in the vQTL analysis.

I analyzed four lipid traits, including high-density lipoprotein cholesterol (HDL), low-density lipoprotein cholesterol (LDL), triglyceride (TG), and total cholesterol (TCHO). Supplementary Figure S16 shows the histograms of the four lipid traits. All four traits are right-skewed with positive skewness values, especially TG (skewness = 2.16). I first removed SNP’s main effect and covariates’ effects from each phenotype. Specifically, I extracted the residuals of regressing each trait on $G_{1,j}$ , $G_{2,j}$ (two indicator variables separating the three genotype groups of a SNP), and 17 covariates, including sex (male vs female), age (in years), BMI (in kg/m^2^), current smoking status (yes vs no), current drinking status (yes vs no), performing physical exercise (yes vs no), educational attainment (integer ranging from 1 to 7), and 10 ancestry PCs. Current smoking was defined as “having smoked cigarettes for at least 6 months when joining the TWB”. Drinking indicated “having a weekly intake of more than 150 mL of alcoholic beverages for at least 6 months when joining the TWB”. Regular exercise meant “performing exercise lasting for 30 min thrice a week”. Educational attainment was coded as an integer ranging from 1 to 7: 1 (illiterate), 2 (no formal education but literate), 3 (primary school graduate), 4 (junior high school graduate), 5 (senior high school graduate), 6 (college graduate), and 7 (Master’s or higher degree).

Sex, age, and BMI significantly influence people’s lipid profiles (Beyene et al., 2020). Smoking impairs lipid metabolism by decreasing LDL receptor expression (Ma et al., 2020). Alcohol consumption disturbs lipid metabolism by increasing adipose tissue lipolysis and causing ectopic fat deposition in the liver (Steiner and Lang, 2017). Regular physical activity has been found to improve lipoprotein-lipid profiles (Lin, 2021). Moreover, lower educational attainment is usually linked to worse lipid profiles (Lara and Amigo, 2018). Because these seven covariates are associated with lipid traits, I adjusted them in all vQTL methods. Furthermore, the first 10 ancestry PCs are adjusted in genetic analyses to avoid population stratification (Uffelmann et al., 2021).

Based on the simulation results, I chose the KW test as the primary vQTL method. This non-parametric approach is robust to outliers by transforming trait values into ranks (Figure 1; Supplementary Figure S1–S3F). Moreover, although KW has a slight inflation in FPR for skewed trait distributions (Figure 1; Supplementary Figure S1–S3K), investigators can perform a follow-up regression analysis to check whether GxE and GxG exist (i.e., the so-called “direct GxE or GxG analysis”). Furthermore, KW needs only 1/70–1/90 of the other non-parametric competitor’s (QUAIL) execution time. Because SNPs with MAFs <0.05 are difficult to replicate in GxG or GxE findings (Lin, 2024; Wang et al., 2019), I only analyzed 2,580,790 common SNPs with MAFs ≥ 0.05.

With the PLINK clumping procedure (Purcell et al., 2007), I detected 10 independent HDL-vQTLs (KW p-value < 5E-8) with linkage disequilibrium (LD) measure *r* ^*2*^ < 0.01. Moreover, 6, 12, and 6 independent vQTLs were identified from LDL, TG, and TCHO, respectively. Four SNPs were found to be vQTLs of multiple lipid traits, including rs4704208 (near *HMGCR*), rs11748027 (in *ANKDD1B*), rs483082 (in *APOC1*), and rs662799 (in *APOA5*). Table 3 summarizes the 30 (=10 + 6+12+6–4) unique vQTLs of the four lipid traits.

**TABLE 3:** Thirty vQTLs (KW p-value < 5E-8 and linkage disequilibrium measure *r* ^*2*^ < 0.01) of the four lipid traits.

Trait (number of vQTLs)	Chromosome	Base pair	vQTL	Gene
HDL (10)	9	104827463	rs4149307	*ABCA1*
	9	104902020	rs1883025	*ABCA1*
	15	58431476	rs1800588	*LIPC*
	16	56957451	rs183130	Near *CETP*
	16	67998971	rs113731015	*DPEP2*
	16	68256297	rs12446418	*PLA2G15*
	18	49627658	rs80208964	*LOC105372112*
	19	11236981	rs737338	*DOCK6*
	19	44912383	rs445925	*APOC1*
	20	45907572	rs148753678	*PLTP*
LDL (6)	2	21024193	rs57825321	*APOB*
	5	75325846	rs4704208^a^	Near *HMGCR*
	5	75614147	rs11748027^b^	*ANKDD1B*
	11	116786951	rs3741297	*ZPR1*
	19	11131631	rs2738464	*LDLR*
	19	44912921	rs483082^c^	*APOC1*
TG (12)	1	62436136	rs631106	*USP1*
	1	62690372	rs1168114	*DOCK7*
	2	27518370	rs780094	*GCKR*
	2	27798099	rs3935148	*RBKS*
	7	73606007	rs3812316	*MLXIPL*
	8	19966163	rs1803924	*LPL*
	8	125465736	rs2001945	Near *TRIB1*
	11	61815236	rs174561	*FADS1*
	11	116792991	rs662799^d^	*APOA5*
	11	117050674	rs1815786	*SIK3*
	17	67966122	rs10445361	*BPTF*
	19	44913484	rs438811	*APOC1*
TCHO (6)	1	109275536	rs3832016	*CELSR2*
	2	21029662	rs13306194	*APOB*
	5	75325846	rs4704208^a^	Near *HMGCR*
	5	75614147	rs11748027^b^	*ANKDD1B*
	11	116792991	rs662799^d^	*APOA5*
	19	44912921	rs483082^c^	*APOC1*

^a^ rs4704208 (near *HMGCR*) is a vQTL of LDL and TCHO.

^b^ rs11748027 (in *ANKDD1B*) is a vQTL of LDL and TCHO.

^c^ rs483082 (in *APOC1*) is a vQTL of LDL and TCHO.

^d^ rs662799 (in *APOA5*) is a vQTL of TG and TCHO.

#### 3.2.2 Direct GxE analysis

**FIGURE 2:**
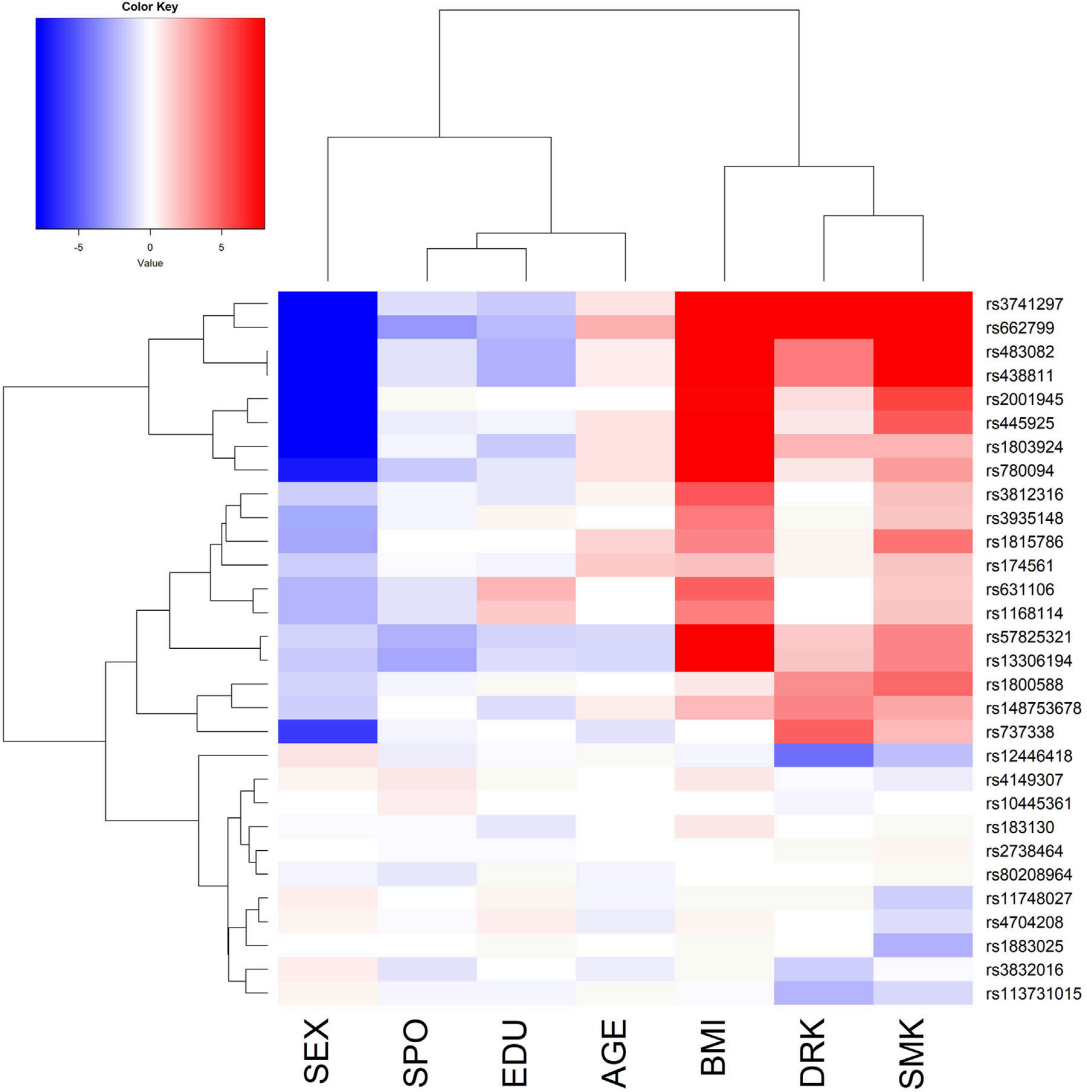
The phylogenetic heat map of the gene-environment interaction analysis for triglyceride (TG). The magnitude of the value represents–log10 (two-sided p-value of the SNP-E interaction), which is always positive. However, I deliberately added a positive/negative sign before the magnitude. A positive sign indicates that the environmental factor (E) exacerbates the vQTLs’ effects. In contrast, a negative sign suggests that the E attenuates the vQTLs’ effects. The *x*-axis lists the 7 Es, including SEX (female vs. male), SPO (performing regular exercise, yes vs no), EDU (educational attainment, an integer from 1 to 7), AGE (chronological age, in years), BMI (in kg/m^2^), DRK (alcohol consumption, yes vs no), and SMK (cigarette smoking status, yes vs no).

From the regression (or variable selection) perspective, it is important to keep the hierarchical structure between main and interaction effects (Zhou et al., 2021). Following previous vQTL research (Wang et al., 2019), after identifying vQTLs, I performed a “direct GxE analysis” by regressing the trait on the minor allele count of each vQTL (0, 1, or 2), seven environmental factors (Es), and the product (interaction) term between the minor allele count and one of the seven Es. To remove population stratification, I also adjusted the top 10 ancestry PCs in this regression. The seven Es are listed as the horizontal axis of Figure 2, including SEX (female vs male), SPO (performing regular exercise, yes vs no), EDU (educational attainment, an integer from 1 to 7), AGE (chronological age, in years), BMI (in kg/m^2^), DRK (alcohol consumption, yes vs no), and SMK (cigarette smoking status, yes vs no). As Westerman et al. (Westerman et al., 2022) indicated, some vQTLs were “pleiotropic” concerning phenotypic variability. Table 2 also shows that four loci are vQTLs shared by multiple lipid traits. Instead of performing the direct GxE and GxG analysis for respective vQTLs of each lipid trait (i.e., 10 vQTLs for HDL, 6 vQTLs for LDL, 12 vQTLs for TG, and 6 vQTLs for TCHO), I analyzed all 30 vQTLs to have a more comprehensive picture of the four lipid traits.

Because TG had more vQTLs compared with the other three traits, I present the phylogenetic heat map of the direct GxE analysis for TG in Figure 2. The *x*-axis of Figure 2 lists the 7 Es. The magnitude of the value in Figure 2 represents–log10 (two-sided p-value of the SNP-E interaction), which is always positive. However, I deliberately added a positive/negative sign before the magnitude. A positive sign indicates that E exacerbates the vQTLs’ effects. In contrast, a negative sign suggests that the E attenuates the vQTLs’ effects. Females have more attenuated TG genetic effects than males (blue color in SEX column), whereas higher BMI, alcohol consumption, and cigarette smoking lead to more substantial TG genetic effects (red color in BMI, DRK, and SMK columns). Moreover, the phylogenetic heat maps for the remaining three traits are demonstrated in Supplementary Figures S17–S19.

#### 3.2.3 Direct GxG analysis

**FIGURE 3:**
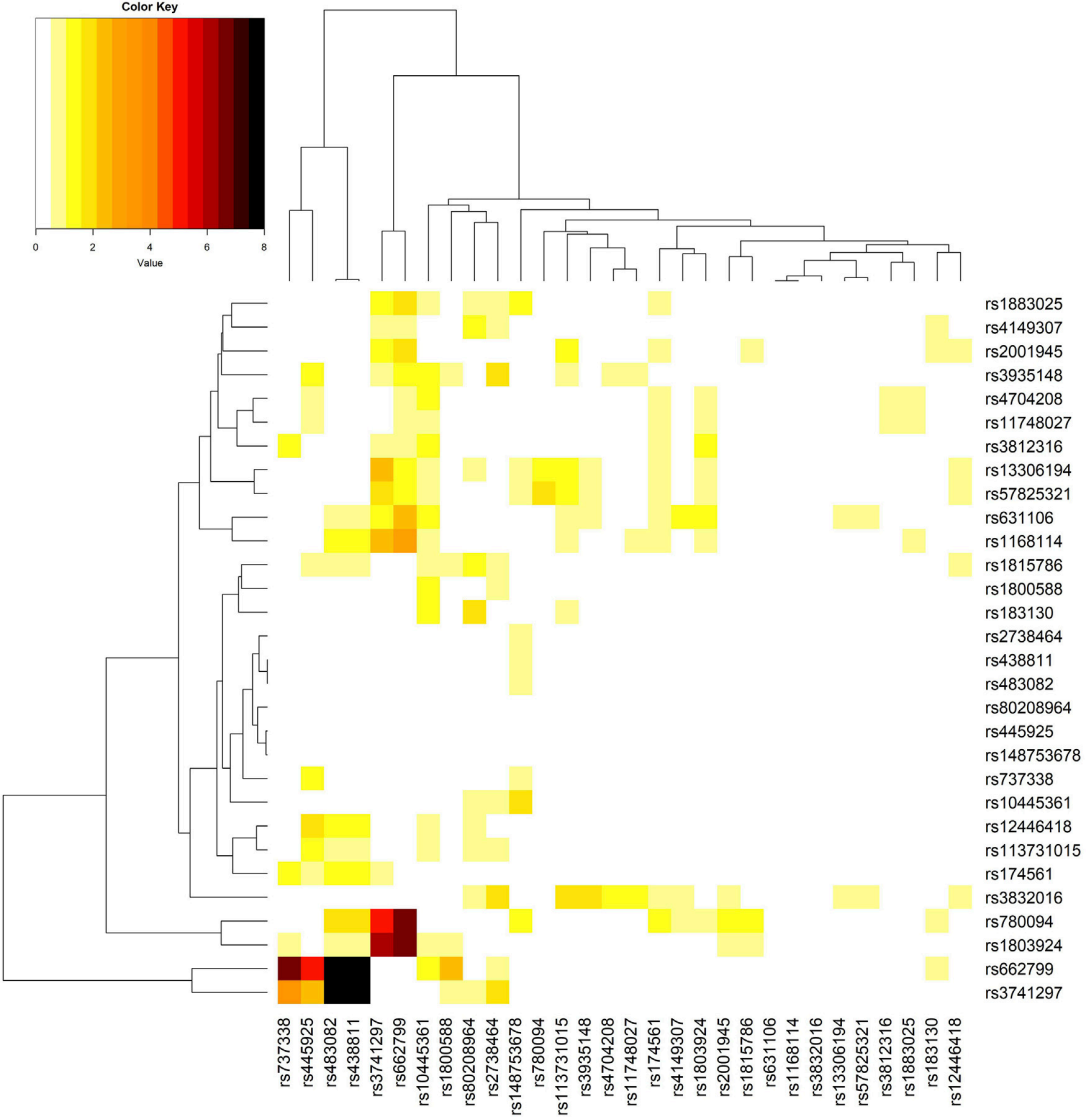
The phylogenetic heat map of the gene-gene interaction analysis for triglyceride (TG). The magnitude of the value represents–log10 (two-sided p-value of the SNP-SNP interaction), which is always positive. The most significant SNP-SNP interaction is between *rs438811* (chr. 19, in *APOC1*) and *rs662799* (chr. 11, in *APOA5*), where p = 1.3E-15. SNP *rs483082* is highly correlated with *rs438811* (*r* ^*2*^ = 0.99), and *rs3741297* is in linkage disequilibrium with *rs662799* (*r* ^*2*^ = 0.19). Therefore, despite four black cells indicating four significant SNP-SNP interactions, I only highlighted *rs438811*-*rs662799* interaction.

I further performed a “direct GxG analysis” by regressing the trait on the minor allele counts (0, 1, or 2) of two SNPs, the seven Es mentioned above, and the product (interaction) term of the minor allele counts of the two SNPs. Similarly, I adjusted the top 10 ancestry PCs in this regression. Figure 3 shows the phylogenetic heat map of the direct GxG analysis for TG. The maps for the other three lipid traits can be found in Supplementary Figures S20–S22.

Regarding TG, the interaction between *rs438811* (chr. 19, in *APOC1*) and *rs662799* (chr. 11, in *APOA5*) is the most significant among the $\left(\begin{array}{c} 30\\ 2 \end{array}\right)=435$ GxG tests constructed by the 30 vQTLs (p = 1.3E-15). The magnitude of the value in Figure 3 represents–log10 (two-sided p-value of the SNP-SNP interaction), which is always positive. The most significant SNP-SNP interaction is between *rs438811* (chr. 19, in *APOC1*) and *rs662799* (chr. 11, in *APOA5*), where p = 1.3E-15. SNP *rs483082* is highly correlated with *rs438811* (*r* ^*2*^ = 0.99), and *rs3741297* is in obvious linkage disequilibrium with *rs662799* (*r* ^*2*^ = 0.19). Therefore, despite four black cells in Figure 3 indicating four significant SNP-SNP interactions, I only highlighted *rs438811*-*rs662799* interaction. As analyzed by the direct GxG regression model, each T allele at *rs438811* is associated with 10.8 mg/dL (p = 7.0E-117), and each G allele at *rs662799* is associated with 26.4 mg/dL (p ∼ 0, extremely low p-value). Possible values of the interaction (product) term between *rs438811* and *rs662799* are 0, 1 (= 1×1 ), 2 (= 1×2 or 2×1 ), and 4 (= 2×2 ). Each point of the interaction term is associated with 5.9 mg/dL (p = 1.3E-15). This result indicates a synergistic interaction between *rs438811* and *rs662799*: T allele at *rs438811* and G allele at *rs662799* working together exerts a more substantial impact on TG than they would have on their own.

## 4 Discussion

Searching for vQTLs greatly facilitates the mining of GxE and GxG. For a GWAS incorporating one million SNPs and seven Es, seven million GxE tests and $\left(\begin{array}{c} 1000000\\ 2 \end{array}\right)=499999500000$ GxG tests are required to investigate GxE and GxG. In the data analysis of this work, 30 vQTLs were identified from four lipid traits. I only needed to perform 210 ( =30×7 ) GxE and 435 ( $=\left(\begin{array}{c} 30\\ 2 \end{array}\right)$ ) GxG tests for each trait. The number of tests has dramatically decreased, and the penalty for multiple tests can be attenuated with a preliminary vQTL search.

This work evaluates the performance of three parametric (DRM, BF, and DGLM) and two non-parametric (KW and QUAIL) methods in detecting vQTLs. Although DGLM is the most powerful method for normally distributed traits (Figure 1; Supplementary Figure S1–S3C–E), it has severe inflation in FPR for traits following other distributions (Figure 1; Supplementary Figure S1–S3F, K). The random error term of DGLM is assumed to follow the normal distribution. This is why it fails to control for FPR when the trait is non-normally distributed. Therefore, DGLM should not be adopted for vQTL detection when the trait distribution is unclear. Taking INT transformation helps adjust FPR for kurtotic traits (Figure 1; Supplementary Figure S1–S3F) but not for skewed traits (Figure 1; Supplementary Figure S1–S3K). Transforming trait values into ranks like INT and KW can address the outlier problem in kurtotic distributions. However, these two rank-based methods, especially DGLM_INT, cannot fully solve the heteroscedastic problem under skewed traits (Figure 1; Supplementary Figure S1–S3K).

BF has inflated FPR under kurtotic traits when MAF <0.2 (Supplementary Figures S5, S8A–C). The BF test is equivalent to the ANOVA *F* test for regressing $D_{ij}$ on the two indicator variables differentiating the three genotype groups of an SNP. Therefore, BF is not a valid test given kurtotic traits and unbalanced sample sizes across three genotype groups (i.e., only a few observations in a particular genotype group, which is a common situation under small MAF). This unbalanced issue can be alleviated given a larger total sample size (*N*). As we can see, the problem of inflated FPR in BF is not so severe when *N* increases to 147,836 (Supplementary Figures S11, S14A–C).

DRM has a power performance similar to that of BF. These two tests are based on a similar strategy. DRM regresses $D_{\left(i\right)j}$ (the deviation between individual *j*’s covariate-adjusted residual and his/her group median) on the minor allele count at each SNP (i.e., 0, 1, or 2), whereas BF regresses $D_{\left(i\right)j}$ on the two indicator variables of genotypes. The only difference between the two methods is that DRM regards genotypes as a continuous scale (coding as 0, 1, and 2), whereas BF treats genotypes as a categorical scale. It is unsurprising that DRM and BF perform similarly in power. However, DRM controls FPR more appropriately than BF, because unbalanced sample size is a more critical issue when separating genotypes into three groups.

Two non-parametric tests, QUAIL and KW, were evaluated in this work. Although QUAIL maintains an appropriate FPR under normally or non-normally distributed traits (column 1 of Figure 1; Supplementary Figure S1–S3), its statistical power is never among the optimal choices under any situation (columns 2–5 of Figure 1; Supplementary Figure S1–S3). Besides, the power of QUAIL can be compromised by quantile crossing, which is a well-known issue when estimating multiple quantiles simultaneously (Bondell et al., 2010). However, the QUAIL R code does not evaluate whether an analysis suffers from quantile crossing. Moreover, QUAIL requires much longer computation time than the other competitors. Therefore, I would not recommend using QUAIL to perform a genome-wide vQTL search. On the other hand, KW is the most powerful vQTL test for kurtotic phenotypes (Figure 1; Supplementary Figure S1–S3G–J). When the traits are skewed, KW presents a slight inflation in FPR (Figure 1; Supplementary Figure S1–S3K). However, the subsequent direct GxE or GxG analysis can further assess the significance of GxE and GxG. Therefore, after considering the computation time, I chose KW to analyze the four lipid traits in the TWB data.

With the comprehensive simulations in this work, DRM and KW are the most recommended parametric and non-parametric vQTL tests, respectively. Unlike DRM (Marderstein et al., 2021), KW has no specific paper to introduce its implementation in detecting vQTLs. Therefore, as a demonstration, I here used KW to analyze the four TWB lipid traits. Other investigators may also choose DRM to perform genome-wide vQTL searches because of its adequate control of FPR and shorter computational time (Table 1). If one applies two or more methods to the same data set, he/she may explore the superiority of each method by comparing the replication rates of different techniques. That is, one can first separate the data set into a discovery set and a replication set, then calculate the replication rates of different methods.

Same with previous vQTL works (Lin, 2024; Wang et al., 2019), I only analyzed common SNPs with MAFs ≥ 0.05. The reason is that GxE is the joint distribution between a SNP and E, and GxG is the joint distribution between two SNPs. Interaction studies are difficult to replicate if an SNP has a small MAF (Lin, 2024). Figures 2, 3 show that GxE and GxG are enriched among vQTLs of the four lipid traits. With more vQTLs identified from TG, it is not surprising that more GxE and GxG can be explored from this trait (GxE: comparing Figure 2 with Supplementary Figures S17–S19; GxG: comparing Figure 3 with Supplementary Figures S20–S22).

For TG, *APOA5 rs662799* has been found to interact with cigarette smoking and alcohol consumption, based on individuals from Korea (Park and Kang, 2020). These interactions were also associated with metabolic syndrome (MetS) by analyzing subjects from Jilin Province of China (Wu et al., 2016). Because TG is one critical component of MetS, both studies (Park and Kang, 2020; Wu et al., 2016) were in line with our analysis results (*APOA5 rs662799* is on the second row of Figure 2).

DRM and KW are the most recommended parametric and non-parametric vQTL methods. QUAIL appropriately preserves the FPR under normally or non-normally distributed traits. However, its power is never among the optimal choices, and its computational time is much longer than the other competitors. BF suffers from severe inflation in FPR when SNP’s MAF <0.2. We may alleviate this inflation in FPR by increasing the sample size to, say, *N* ∼ 150,000. DRM and BF are based on a similar strategy. DRM regresses the deviation between individuals’ covariate-adjusted residuals and their group medians on the minor allele count of an SNP, whereas BF regresses the deviation between individuals’ covariate-adjusted residuals and their group medians on two indicator variables categorizing three genotypes. Therefore, DRM and BF have similar power. However, BF suffers from more inflation in FPR than DRM does, mainly when a small sample size is observed in one genotype. Although DGLM is the most powerful method given normally distributed traits, it is not valid (i.e., producing large FPR) for non-normally distributed traits. Adopting the rank-based INT transformation can address the outlier problem and adjust the FPR for kurtotic traits. However, INT cannot fully solve the heteroscedastic issue of skewed traits.

Recently, a robust Bayesian mixed model rooted in the one-way ANOVA has been developed (Fan et al., 2025). By specifying a likelihood based on a heavy-tailed distribution, this method can improve robustness in GxE detection. With Fan et al.‘s concept (Fan et al., 2025), the non-robust parametric tests can build a robust mixed-effect model.

## Data Availability

The datasets presented in this study can be found in online repositories. The names of the repository/repositories and accession number(s) can be found below: https://www.twbiobank.org.tw/new_web/, TWBR10810-07.
