# Supplementary material for: Mining for gene-environment and gene-gene interactions: parametric and non-parametric tests for detecting variance quantitative trait loci
Source: Front Genet. 2025 Sep 1;16:1617504. doi: 10.3389/fgene.2025.1617504 (PMC12434048; doi:10.3389/fgene.2025.1617504)
Supplement: Supplementary file 1 [file DataSheet1.pdf]

Supplementary Materials of

Mining for gene-environment and gene-gene interactions: parametric and non-parametric tests for detecting variance quantitative trait loci

Short title: Detecting variance quantitative trait loci

Author

Wan-Yu Lin,[1, 2] *

Affiliations

[1] Institute of Health Data Analytics and Statistics, College of Public Health, National Taiwan University, Taipei, Taiwan

[2] Master of Public Health Program, College of Public Health, National Taiwan University, Taipei, Taiwan

* Corresponding author: Wan-Yu Lin, Ph.D. (linwy@ntu.edu.tw)

Wan-Yu Lin, Ph.D.

Room 501, No. 17, Xu-Zhou Road, Taipei 100, Taiwan

Phone/Fax: +886-2-33668106; E-mail: linwy@ntu.edu.tw

https://orcid.org/0000-0002-3385-4702

Contents

Figure S1. QQ plots (the left column) and power (α = 5E-8; columns 2-5) for vQTL tests (*N* = 30000; without SNP main effects) 3
Figure S2. QQ plots (the left column) and power (α = 5E-8; columns 2-5) for vQTL tests (*N* = 147836; with SNP main effects) 4
Figure S3. QQ plots (the left column) and power (α = 5E-8; columns 2-5) for vQTL tests (*N* = 147836; without SNP main effects) 5
Figure S4. QQ plots when the error term follows a standard normal distribution (*N* = 30000; with SNP main effects) 6
Figure S5. QQ plots when the error term follows a *t* distribution with the degrees of freedom 3 (*N* = 30000; with SNP main effects) 7
Figure S6. QQ plots when the error term follows a chi-square distribution with the degrees of freedom 6 (*N* = 30000; with SNP main effects) 8
Figure S7. QQ plots when the error term follows a standard normal distribution (*N* = 30000; without SNP main effects) 9
Figure S8. QQ plots when the error term follows a *t* distribution with the degrees of freedom 3 (*N* = 30000; without SNP main effects) 10
Figure S9. QQ plots when the error term follows a chi-square distribution with the degrees of freedom 6 (*N* = 30000; without SNP main effects) 11
Figure S10. QQ plots when the error term follows a standard normal distribution (*N* = 147836; with SNP main effects) 12
Figure S11. QQ plots when the error term follows a *t* distribution with the degrees of freedom 3 (*N* = 147836; with SNP main effects) 13
Figure S12. QQ plots when the error term follows a chi-square distribution with the degrees of freedom 6 (*N* = 147836; with SNP main effects) 14
Figure S13. QQ plots when the error term follows a standard normal distribution (*N* = 147836; without SNP main effects) 15
Figure S14. QQ plots when the error term follows a *t* distribution with the degrees of freedom 3 (*N* = 147836; without SNP main effects) 16
Figure S15. QQ plots when the error term follows a chi-square distribution with the degrees of freedom 6 (*N* = 147836; without SNP main effects) 17
Figure S16. Histograms of the four lipid traits 18
Figure S17. The phylogenetic heat map of the gene-environment interaction analysis for high-density lipoprotein cholesterol (HDL) 19
Figure S18. The phylogenetic heat map of the gene-environment interaction analysis for low-density lipoprotein cholesterol (LDL) 20
Figure S19. The phylogenetic heat map of the gene-environment interaction analysis for total cholesterol (TCHO) 21
Figure S20. The phylogenetic heat map of the gene-gene interaction analysis for high-density lipoprotein cholesterol (HDL) 22
Figure S21. The phylogenetic heat map of the gene-gene interaction analysis for low-density lipoprotein cholesterol (LDL) 23
Figure S22. The phylogenetic heat map of the gene-gene interaction analysis for total cholesterol (TCHO) 24

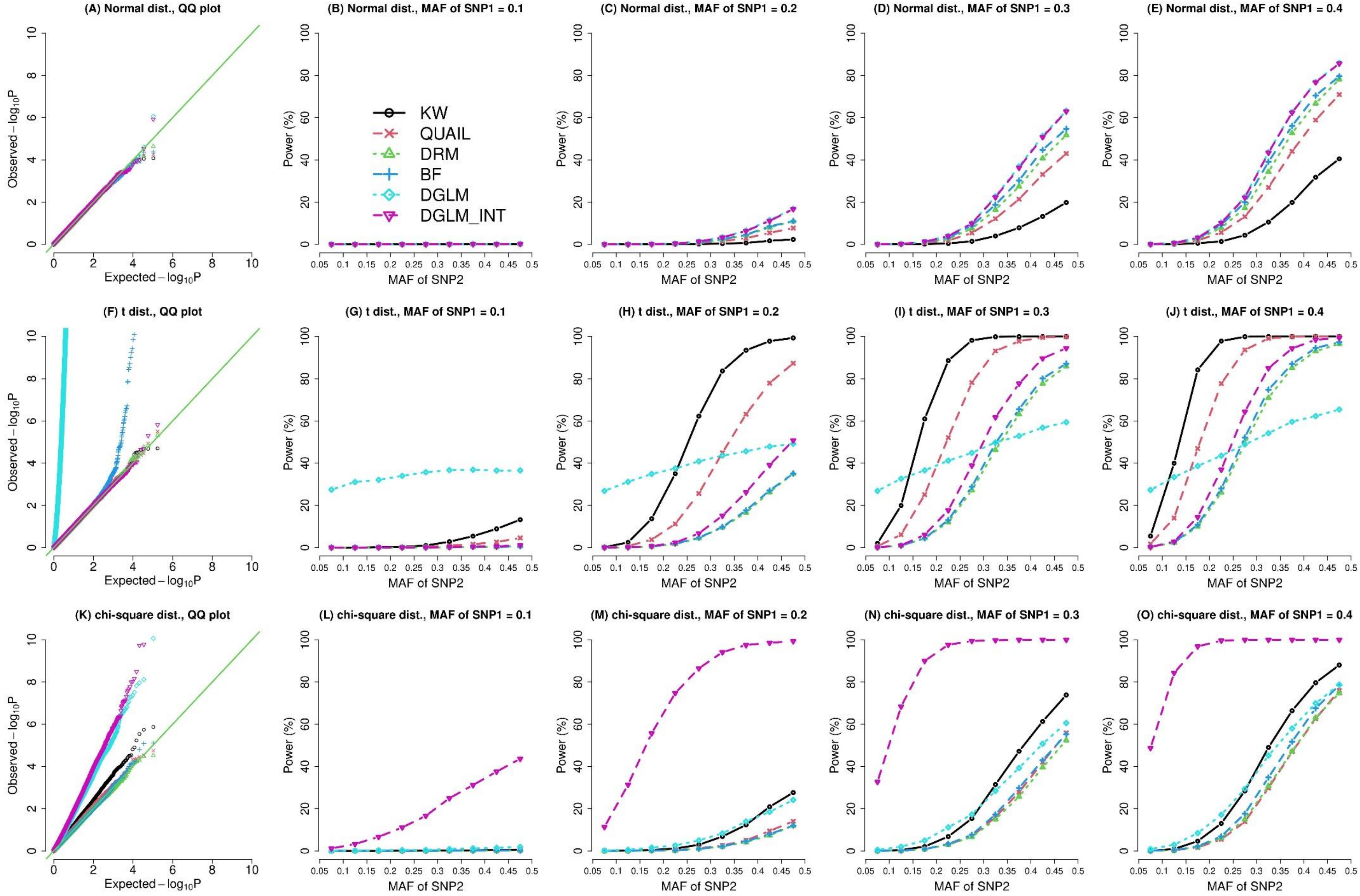

Figure S1. QQ plots (the left column) and power (α = 5E-8; columns 2-5) for vQTL tests (*N* = 30000; without SNP main effects)

The distribution for the error term: (top row) a standard normal distribution; (middle row) a *t* distribution with the degrees of freedom 3; (bottom row) a chi-square distribution with the degrees of freedom 6.

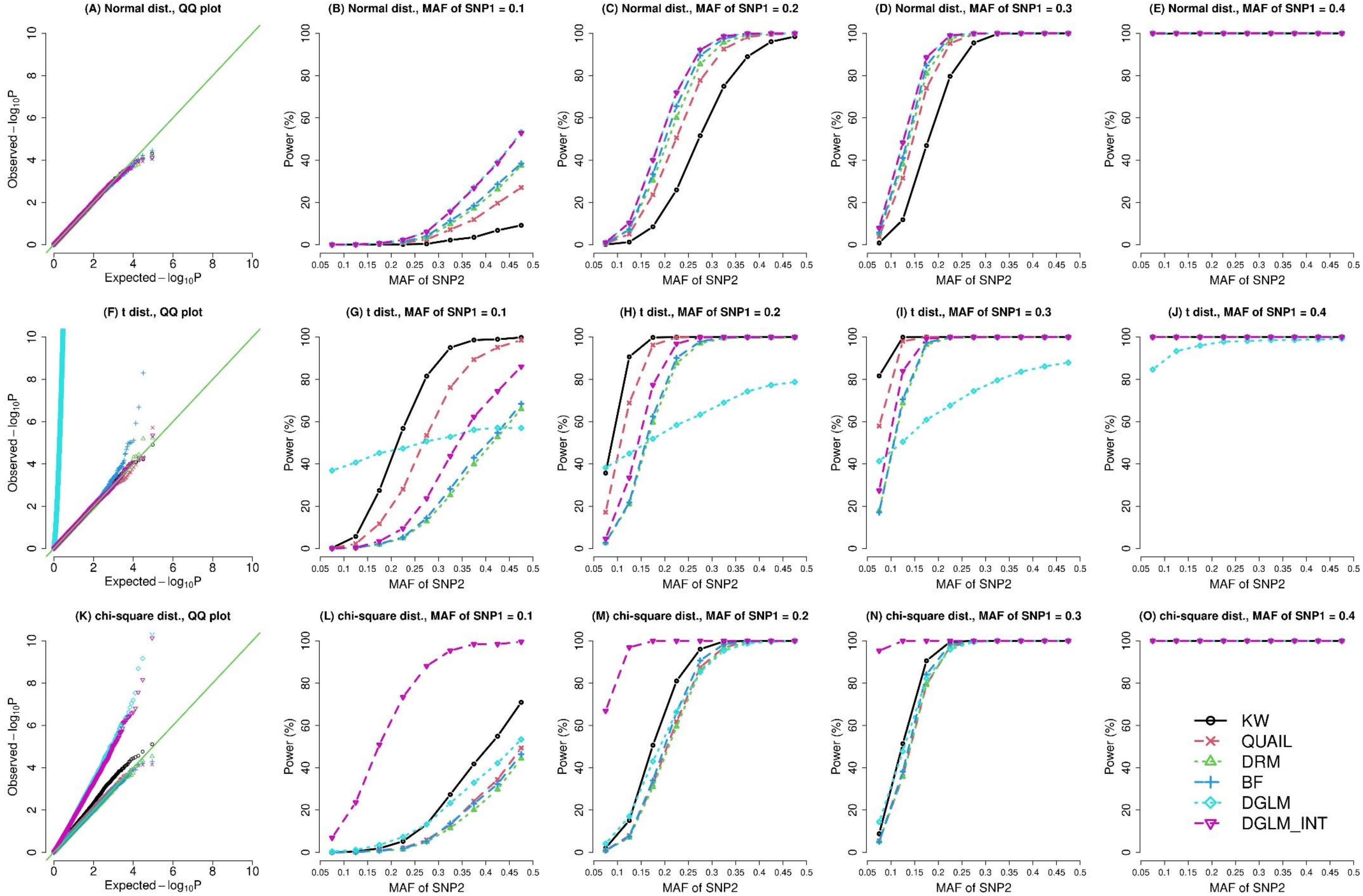

Figure S2. QQ plots (the left column) and power (α = 5E-8; columns 2-5) for vQTL tests (*N* = 147836; with SNP main effects)

The distribution for the error term: (top row) a standard normal distribution; (middle row) a *t* distribution with the degrees of freedom 3; (bottom row) a chi-square distribution with the degrees of freedom 6.

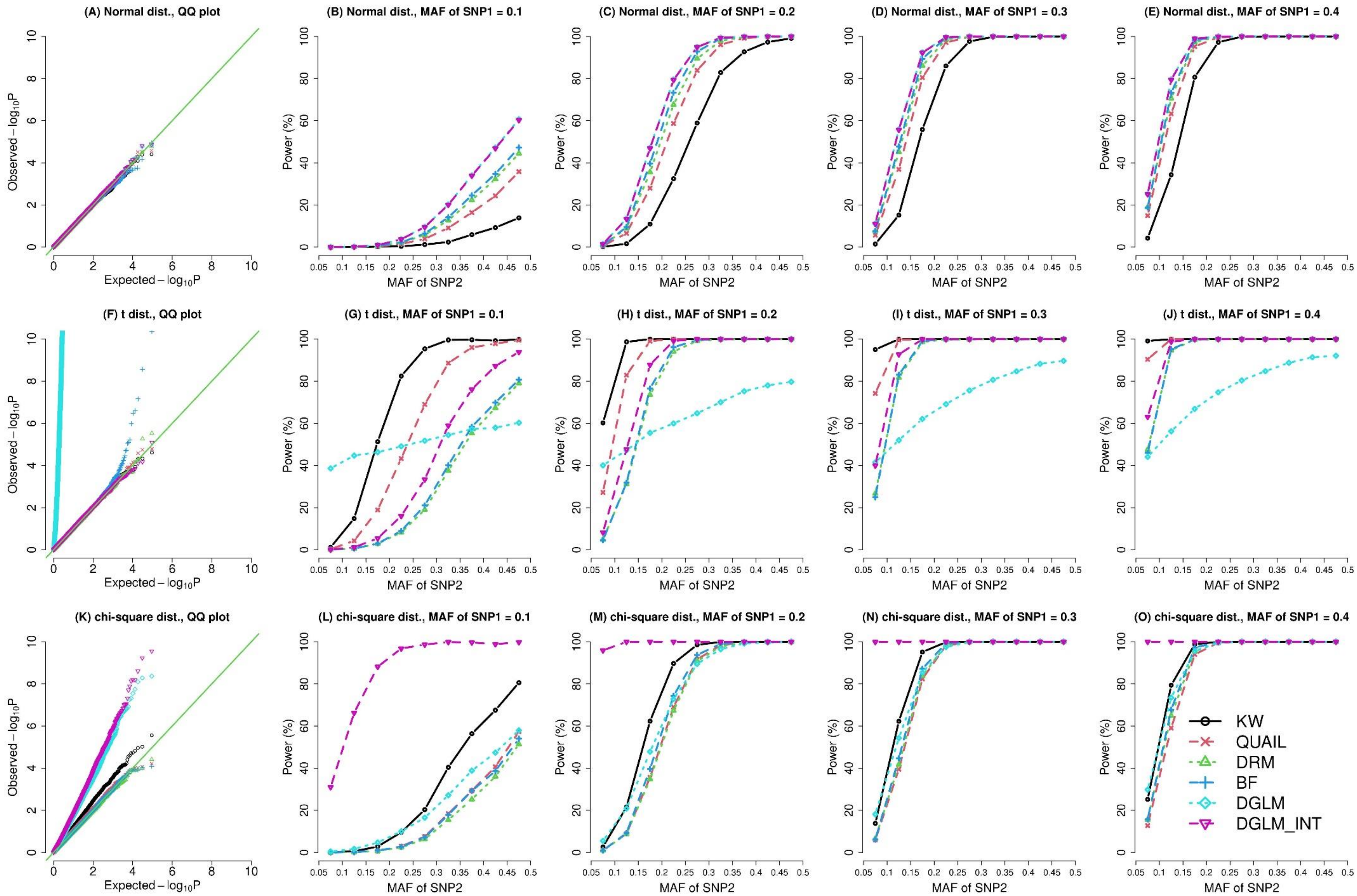

Figure S3. QQ plots (the left column) and power (α = 5E-8; columns 2-5) for vQTL tests (*N* = 147836; without SNP main effects)

The distribution for the error term: (top row) a standard normal distribution; (middle row) a *t* distribution with the degrees of freedom 3; (bottom row) a chi-square distribution with the degrees of freedom 6.

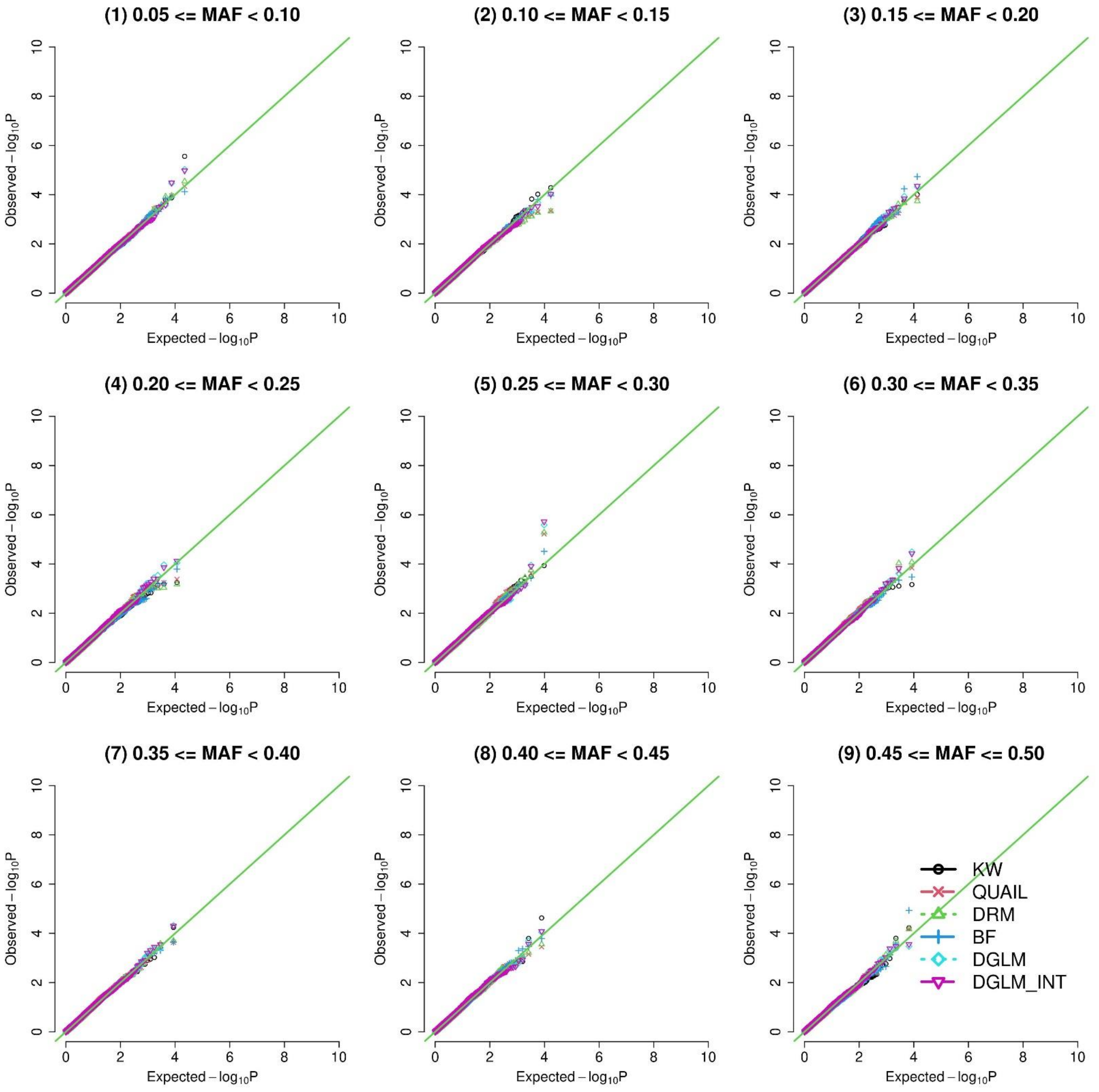

Figure S4. QQ plots when the error term follows a standard normal distribution (*N* = 30000; with SNP main effects)

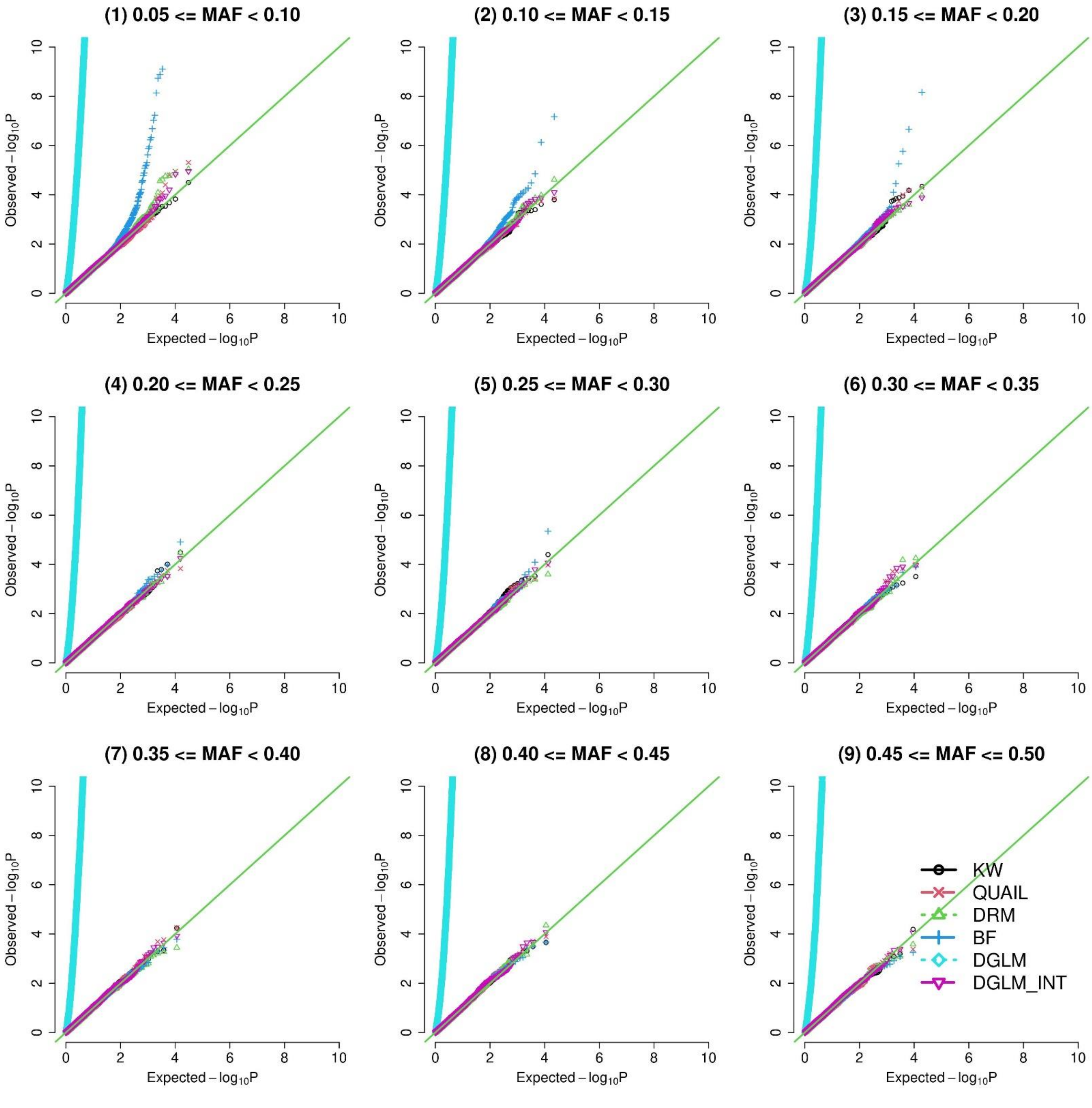

Figure S5. QQ plots when the error term follows a *t* distribution with the degrees of freedom 3 (*N* = 30000; with SNP main effects)

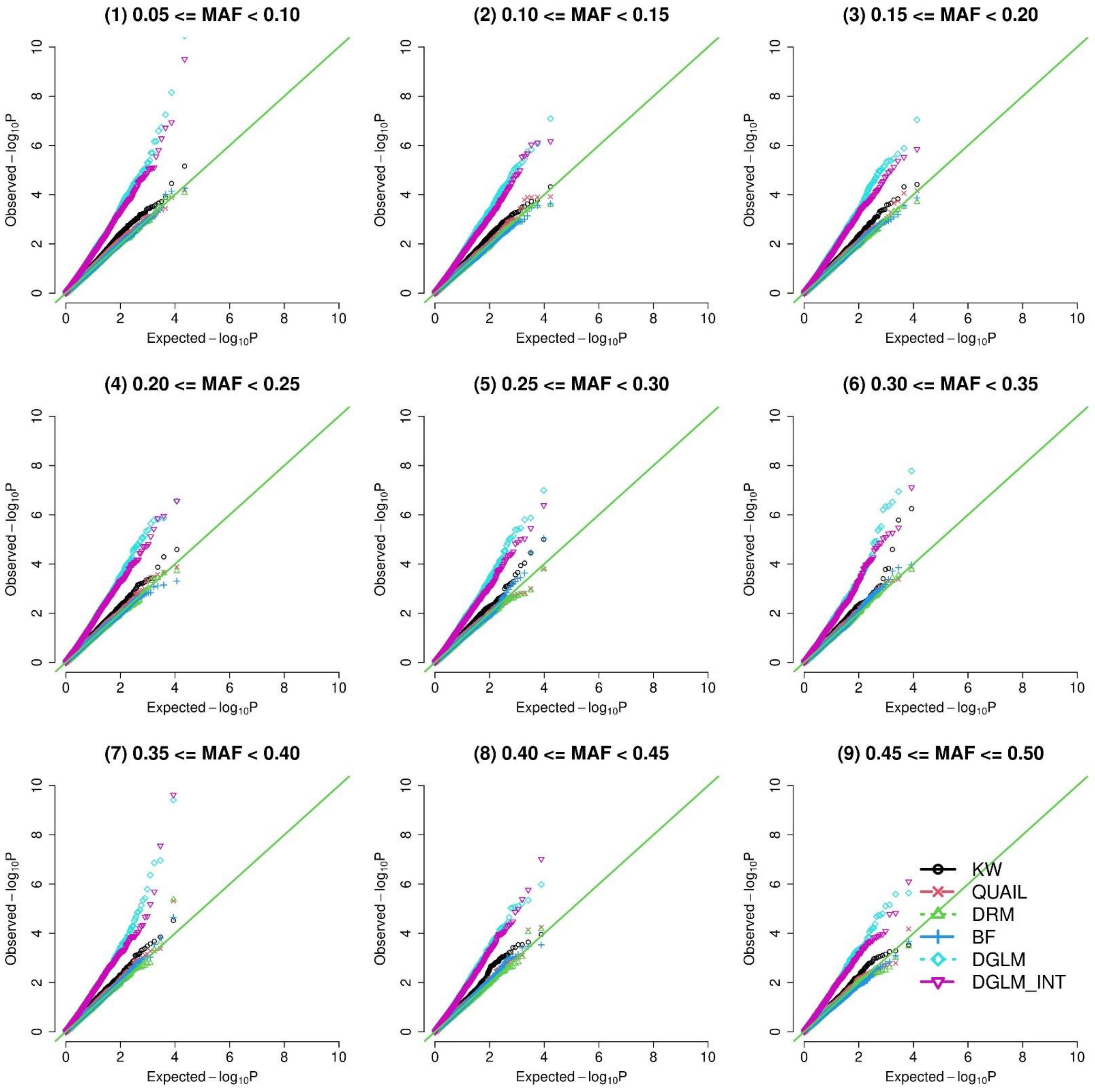

Figure S6. QQ plots when the error term follows a chi-square distribution with the degrees of freedom 6 (*N* = 30000; with SNP main effects)

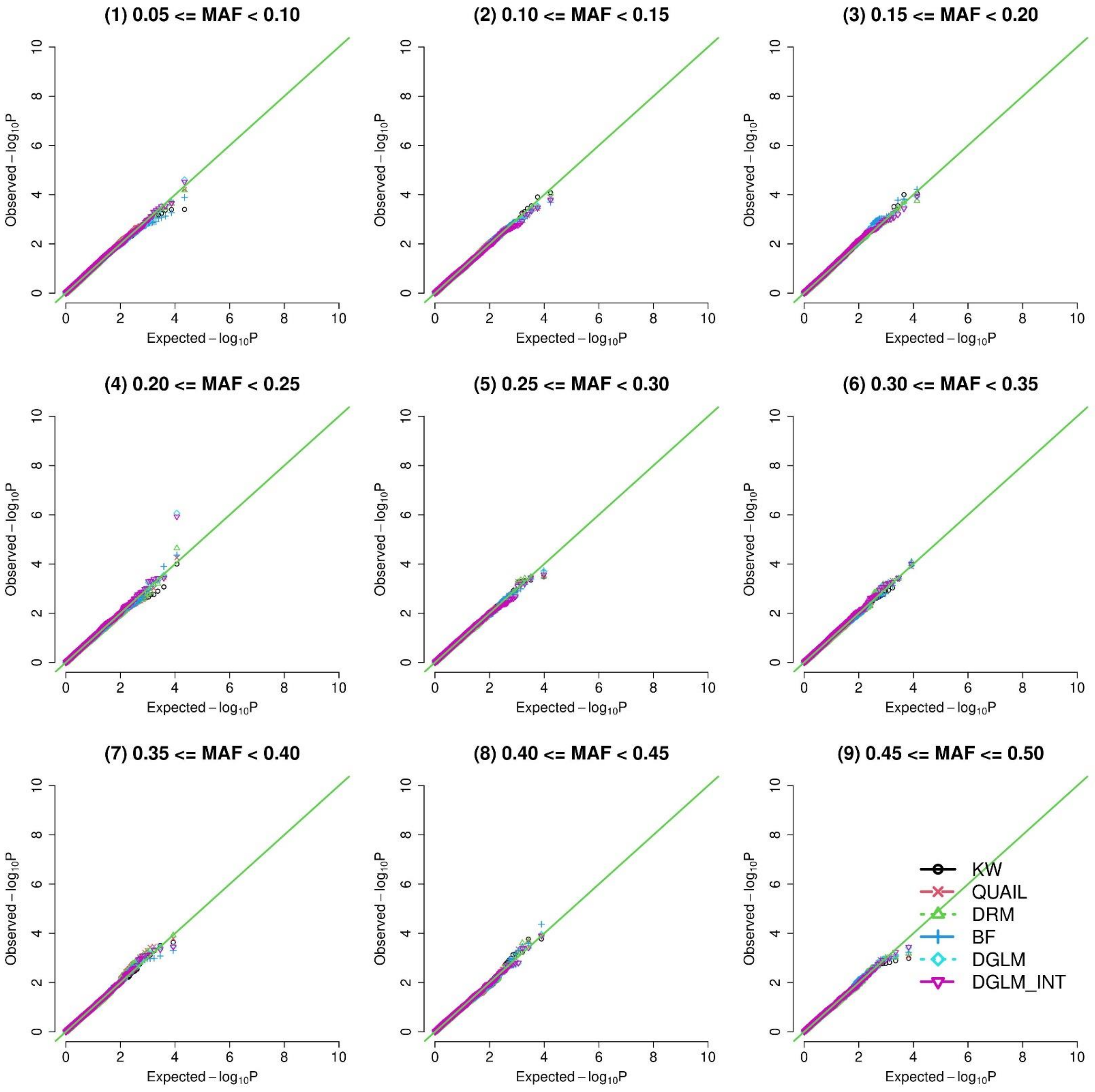

Figure S7. QQ plots when the error term follows a standard normal distribution (*N* = 30000; without SNP main effects)

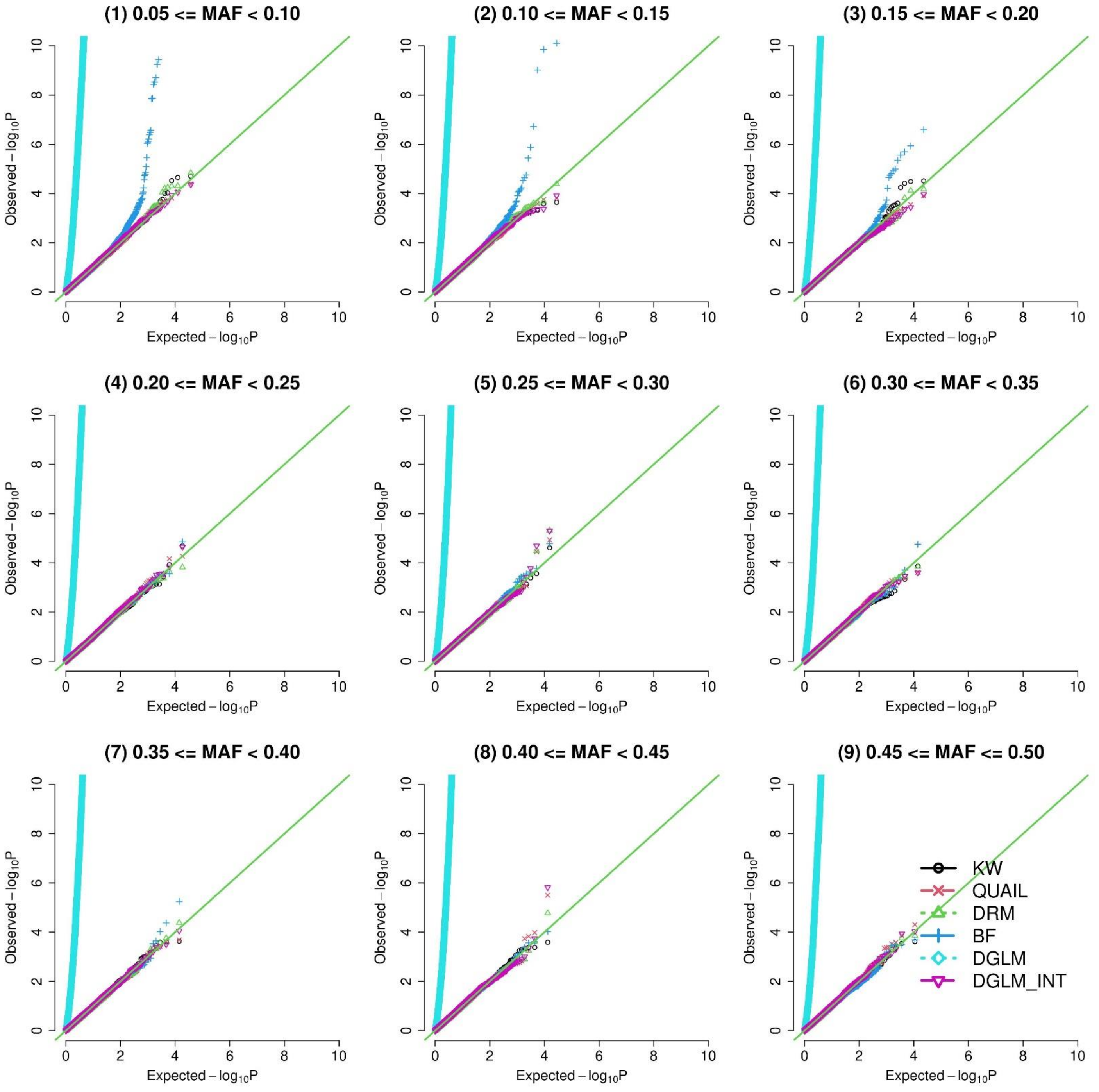

Figure S8. QQ plots when the error term follows a *t* distribution with the degrees of freedom 3 (*N* = 30000; without SNP main effects)

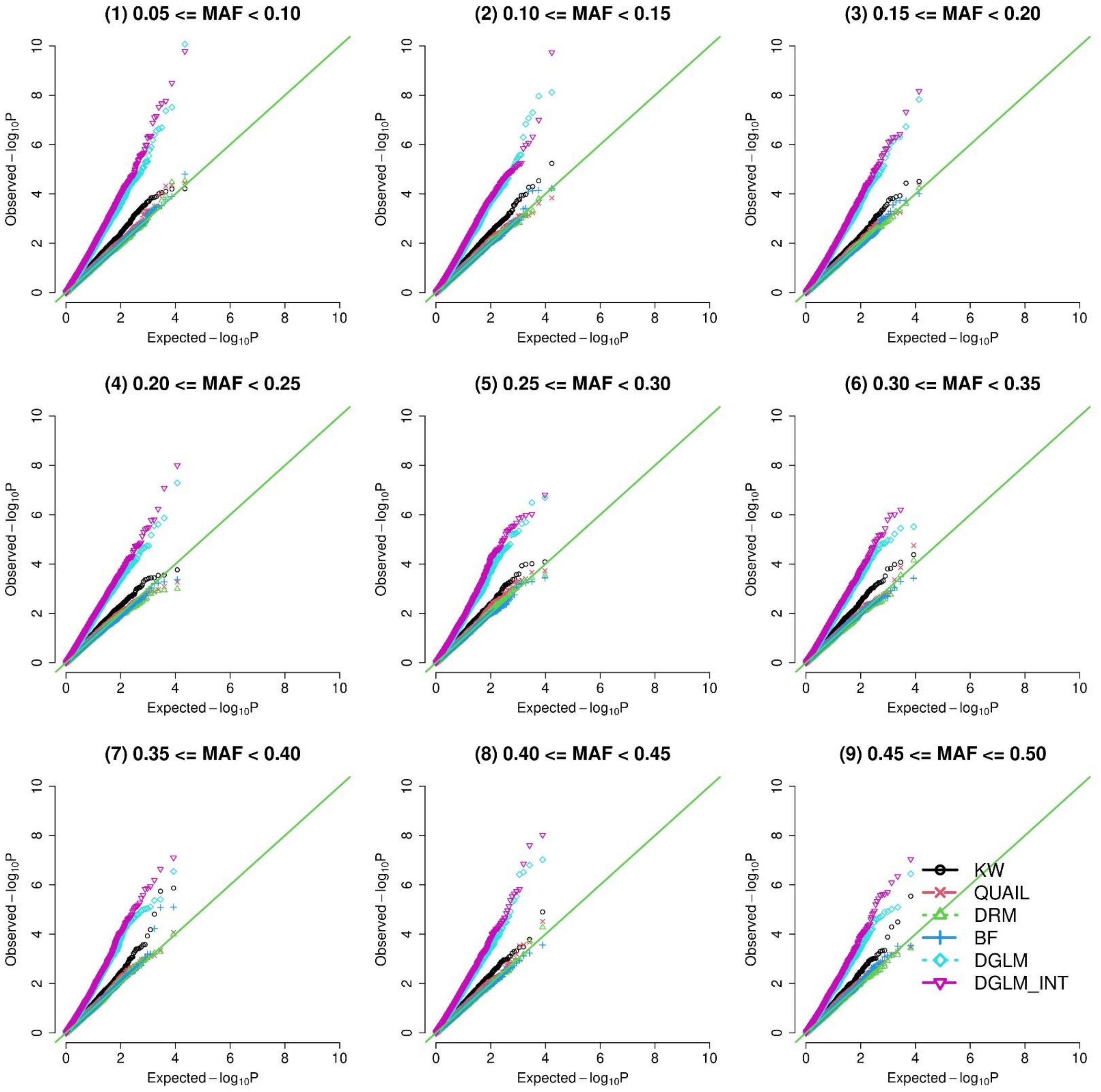

Figure S9. QQ plots when the error term follows a chi-square distribution with the degrees of freedom 6 (*N* = 30000; without SNP main effects)

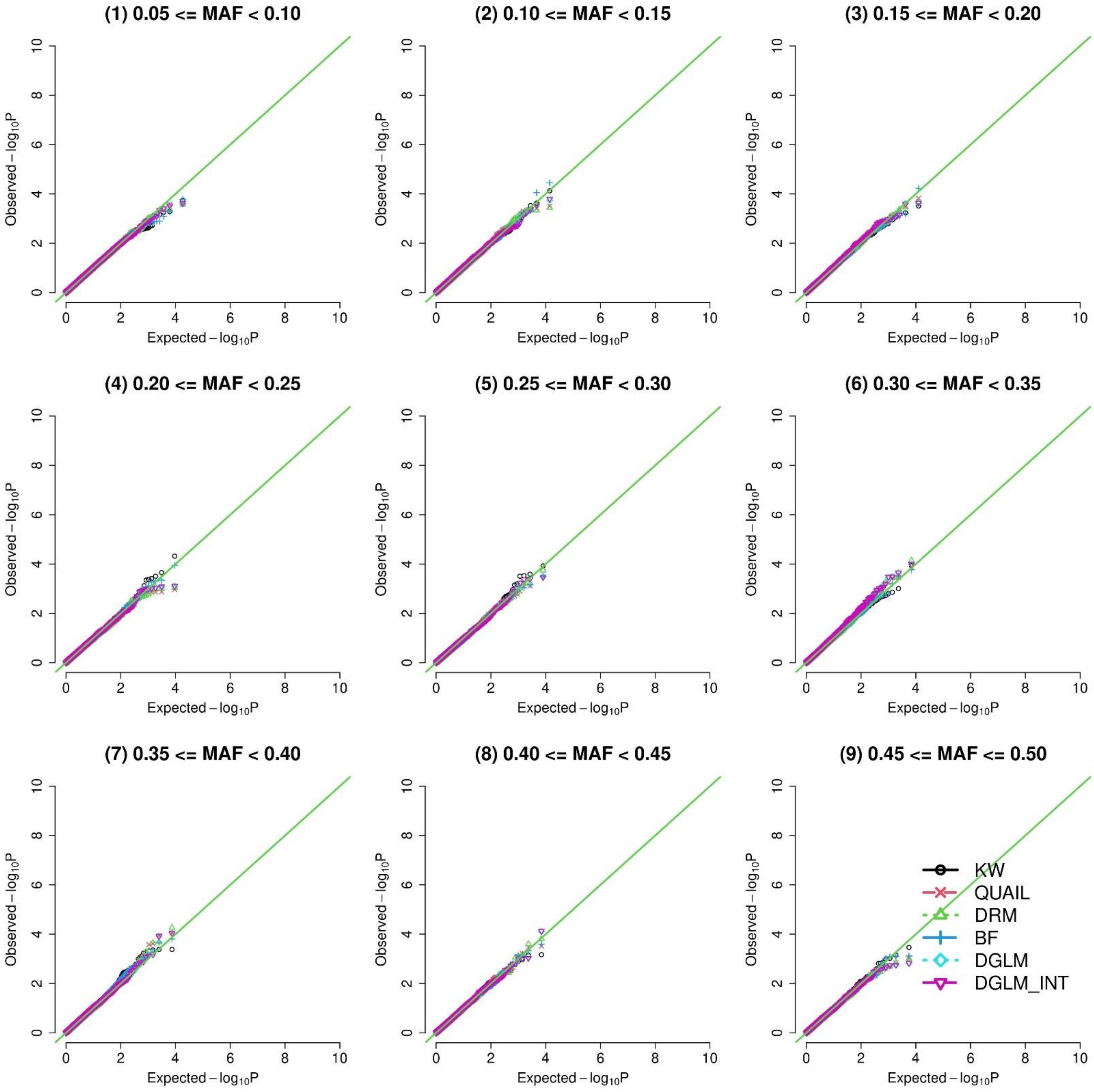

Figure S10. QQ plots when the error term follows a standard normal distribution (*N* = 147836; with SNP main effects)

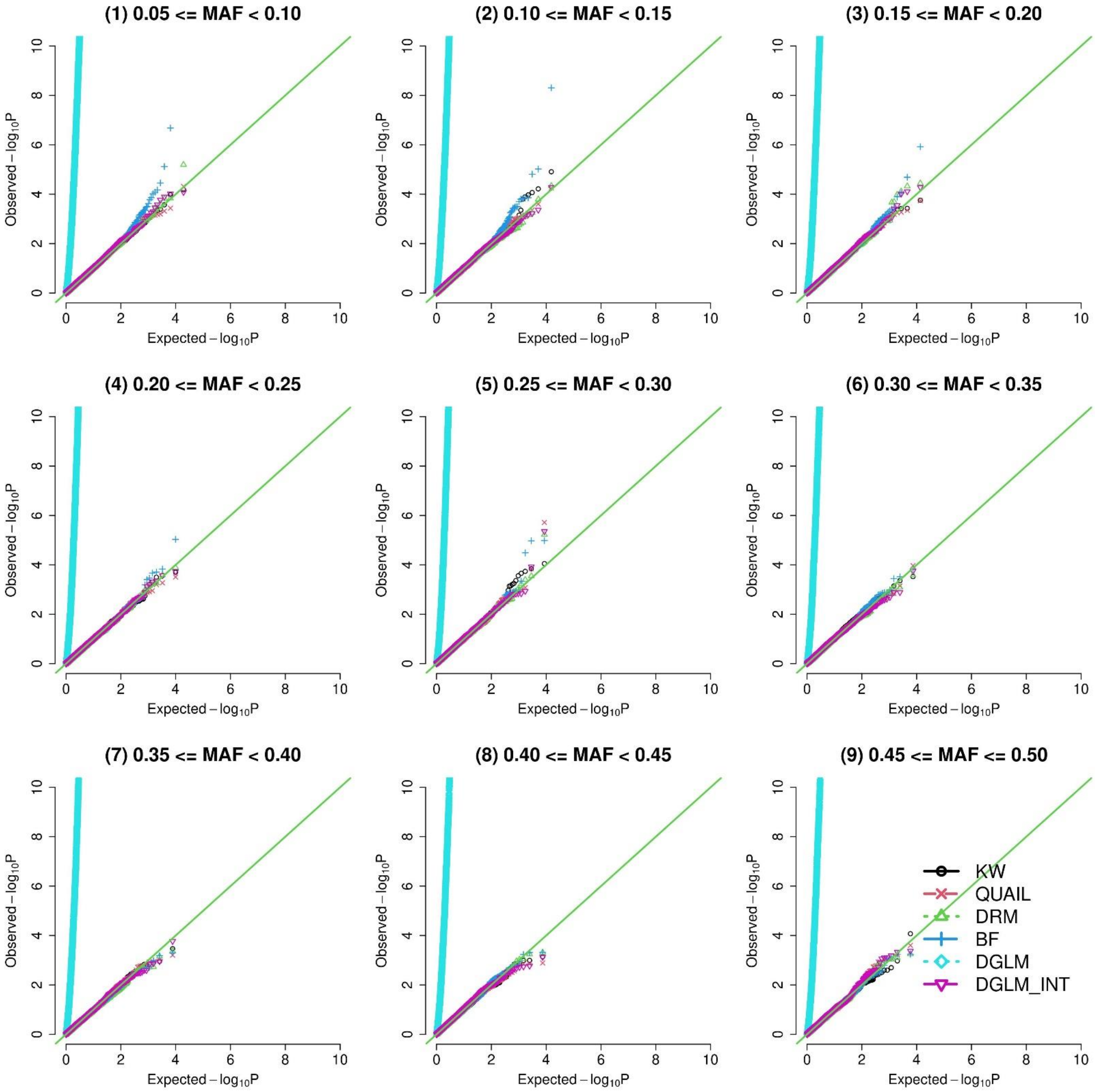

Figure S11. QQ plots when the error term follows a *t* distribution with the degrees of freedom 3 (*N* = 147836; with SNP main effects)

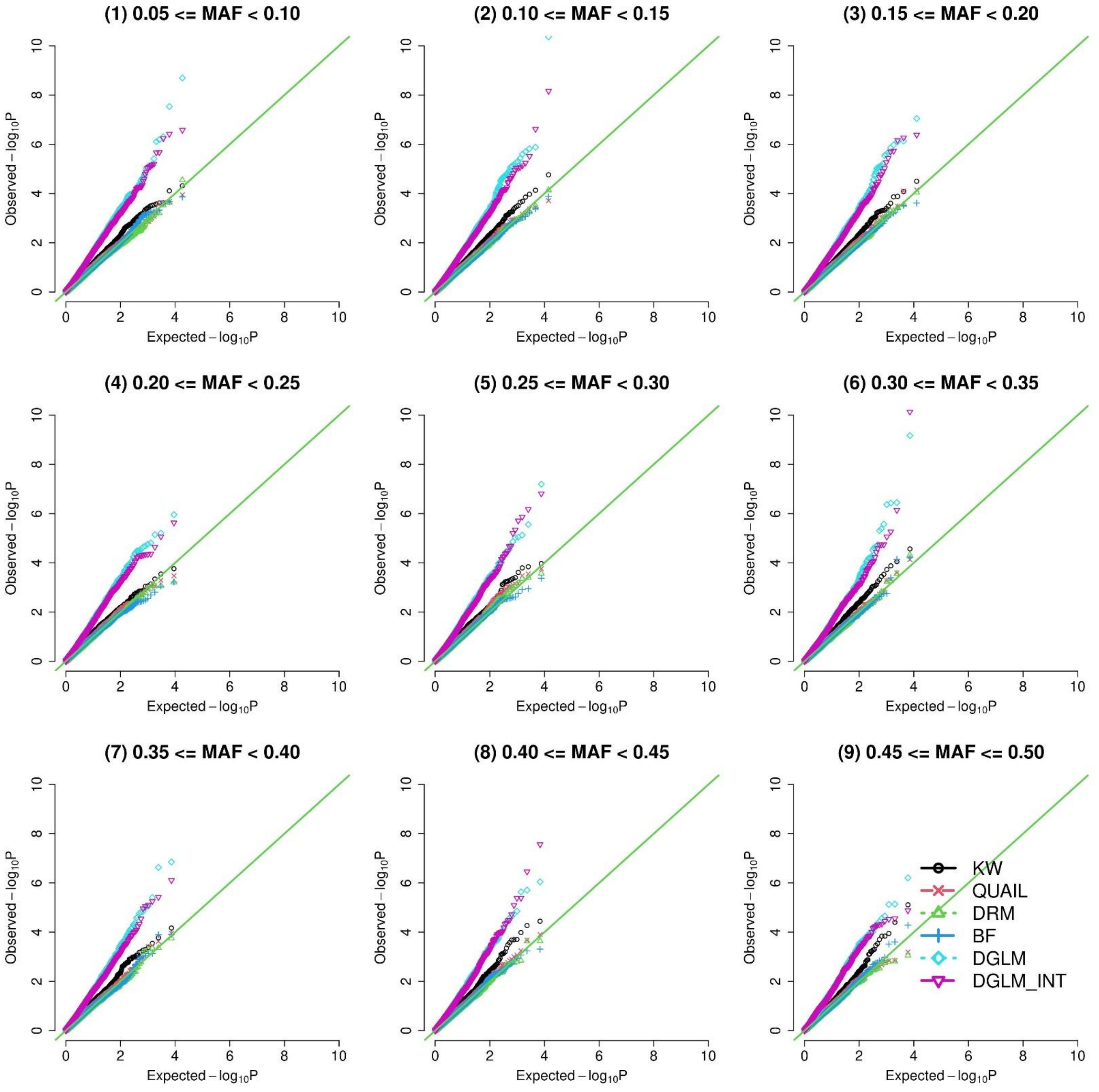

Figure S12. QQ plots when the error term follows a chi-square distribution with the degrees of freedom 6 (*N* = 147836; with SNP main effects)

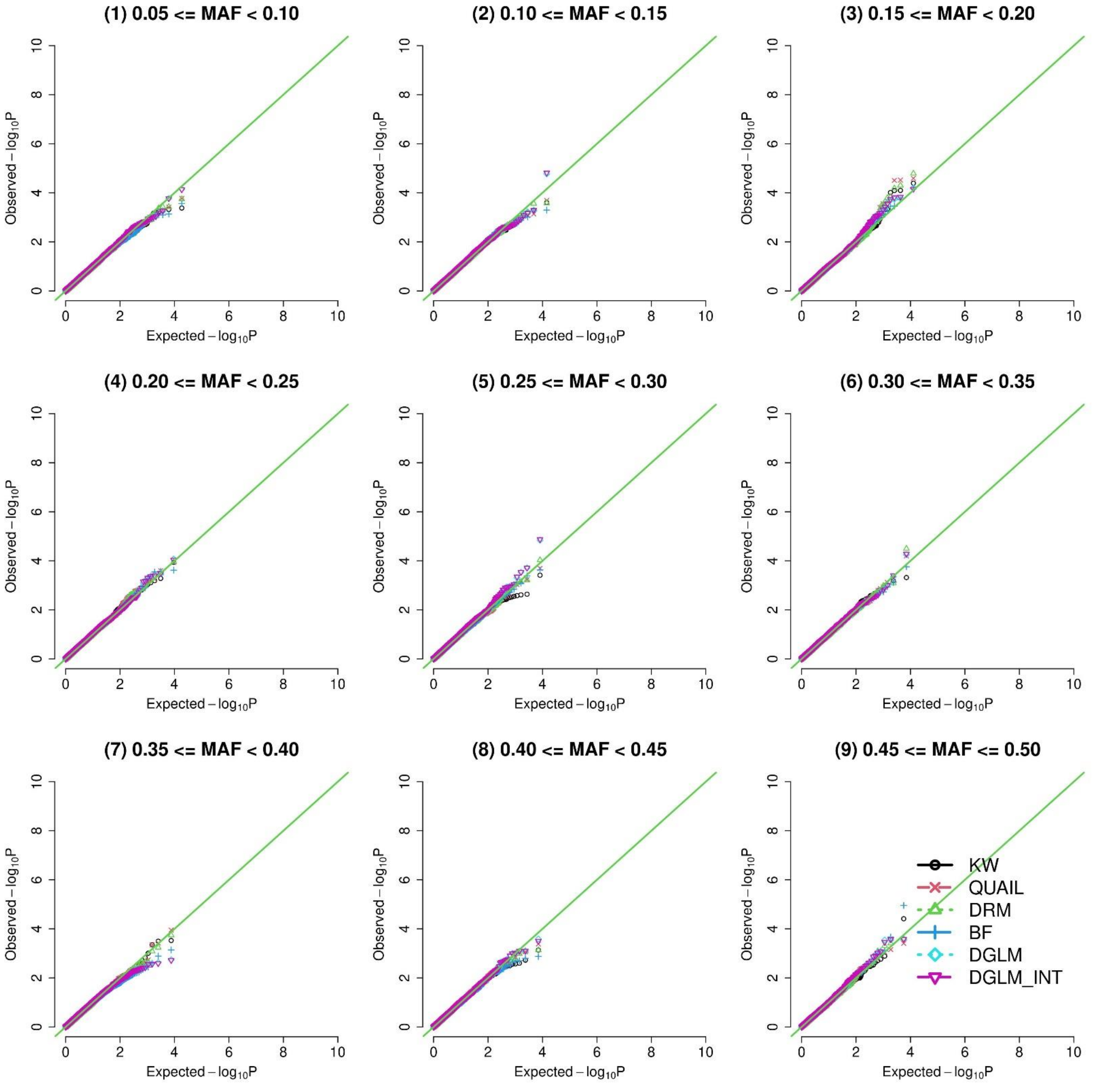

Figure S13. QQ plots when the error term follows a standard normal distribution (*N* = 147836; without SNP main effects)

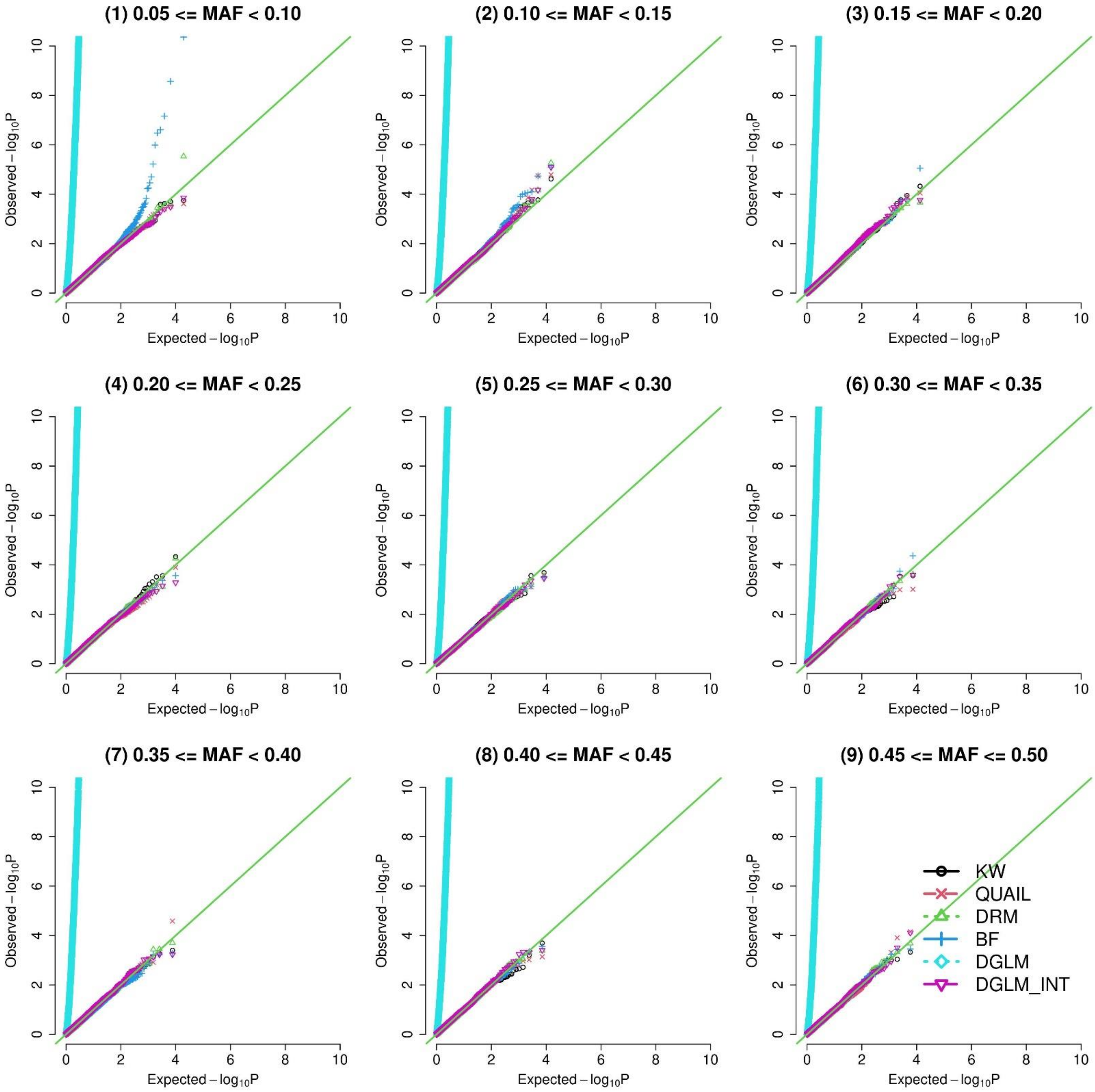

Figure S14. QQ plots when the error term follows a *t* distribution with the degrees of freedom 3 (*N* = 147836; without SNP main effects)

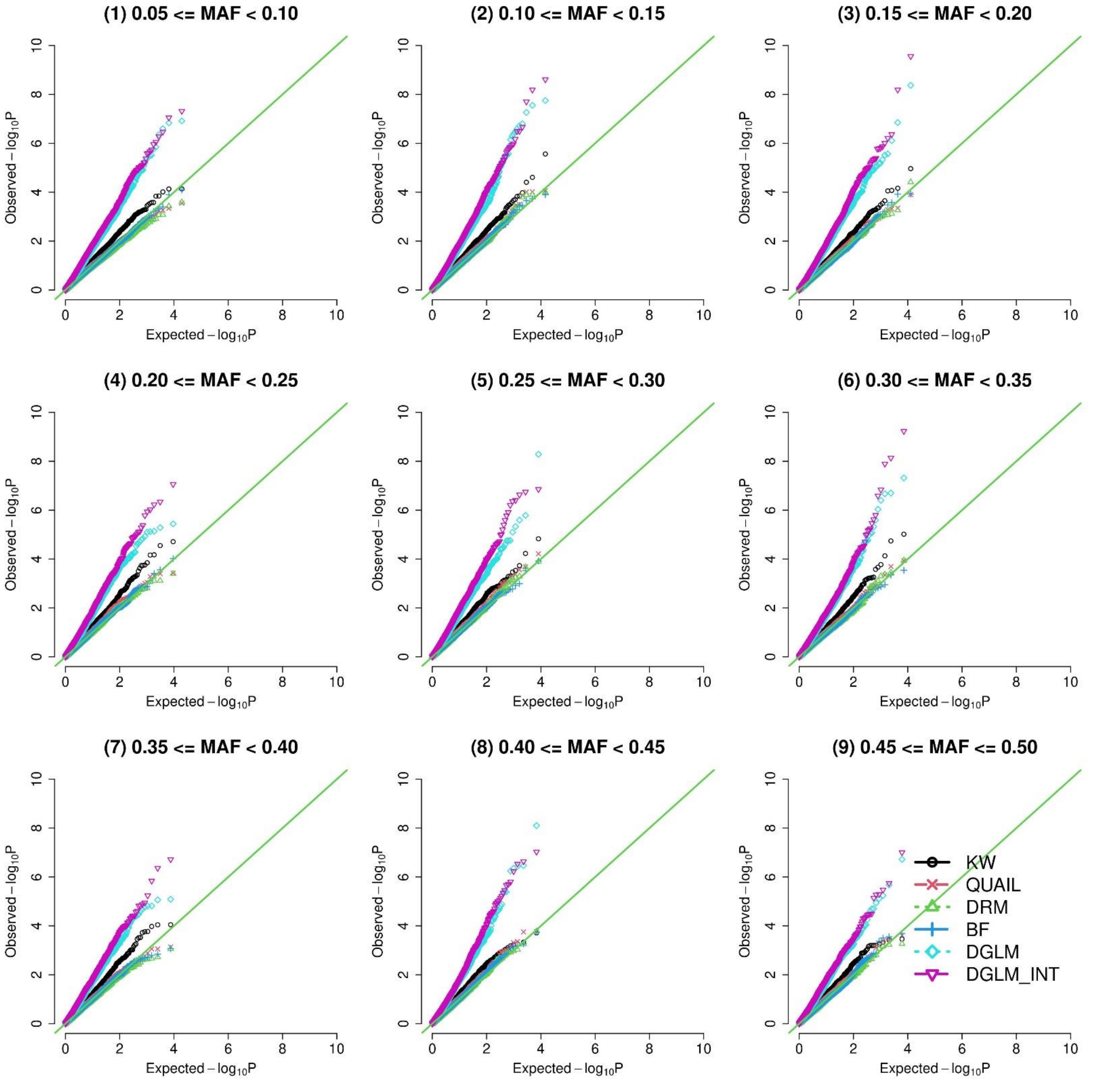

Figure S15. QQ plots when the error term follows a chi-square distribution with the degrees of freedom 6 (*N* = 147836; without SNP main effects)

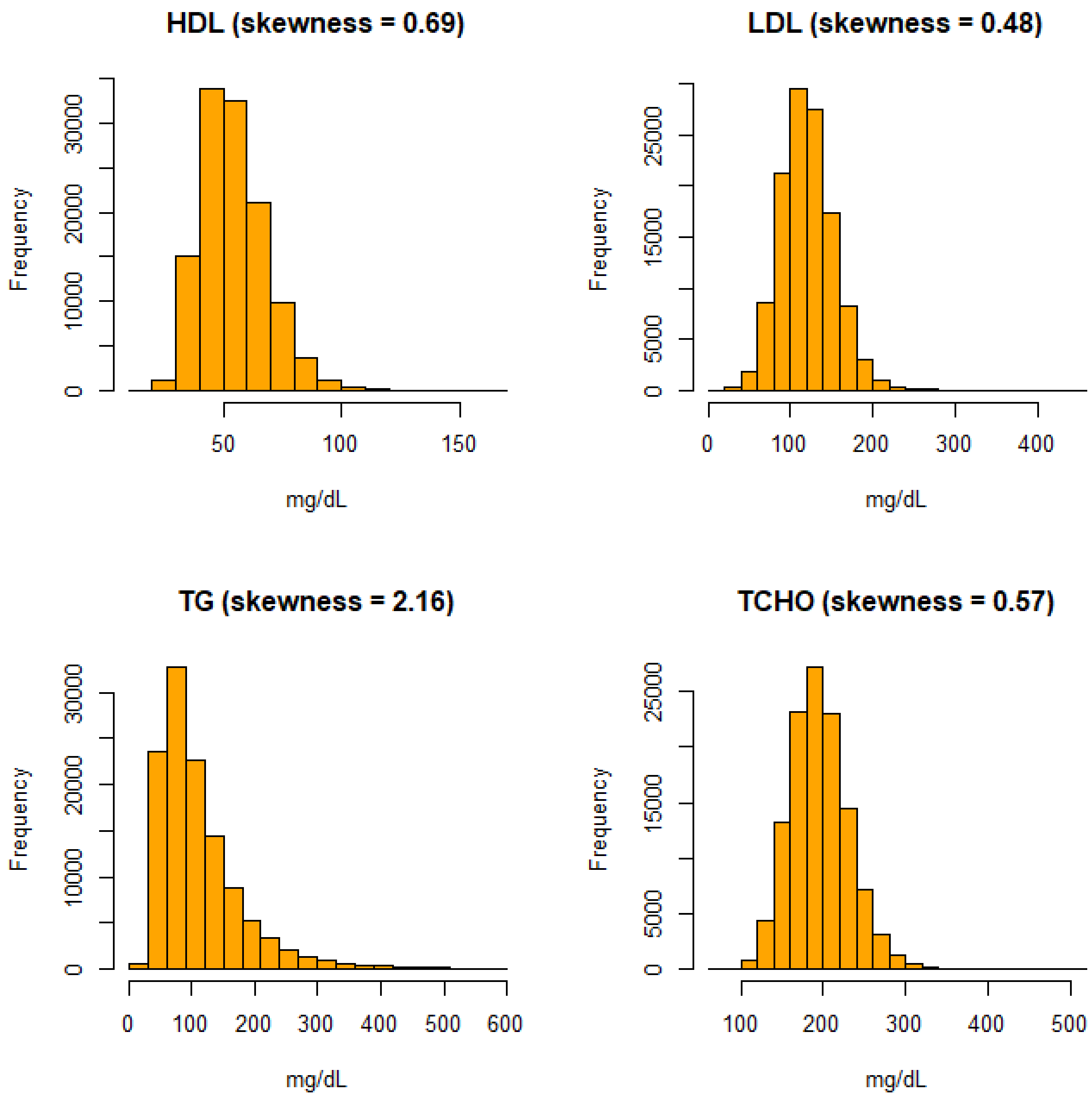

Figure S16. Histograms of the four lipid traits

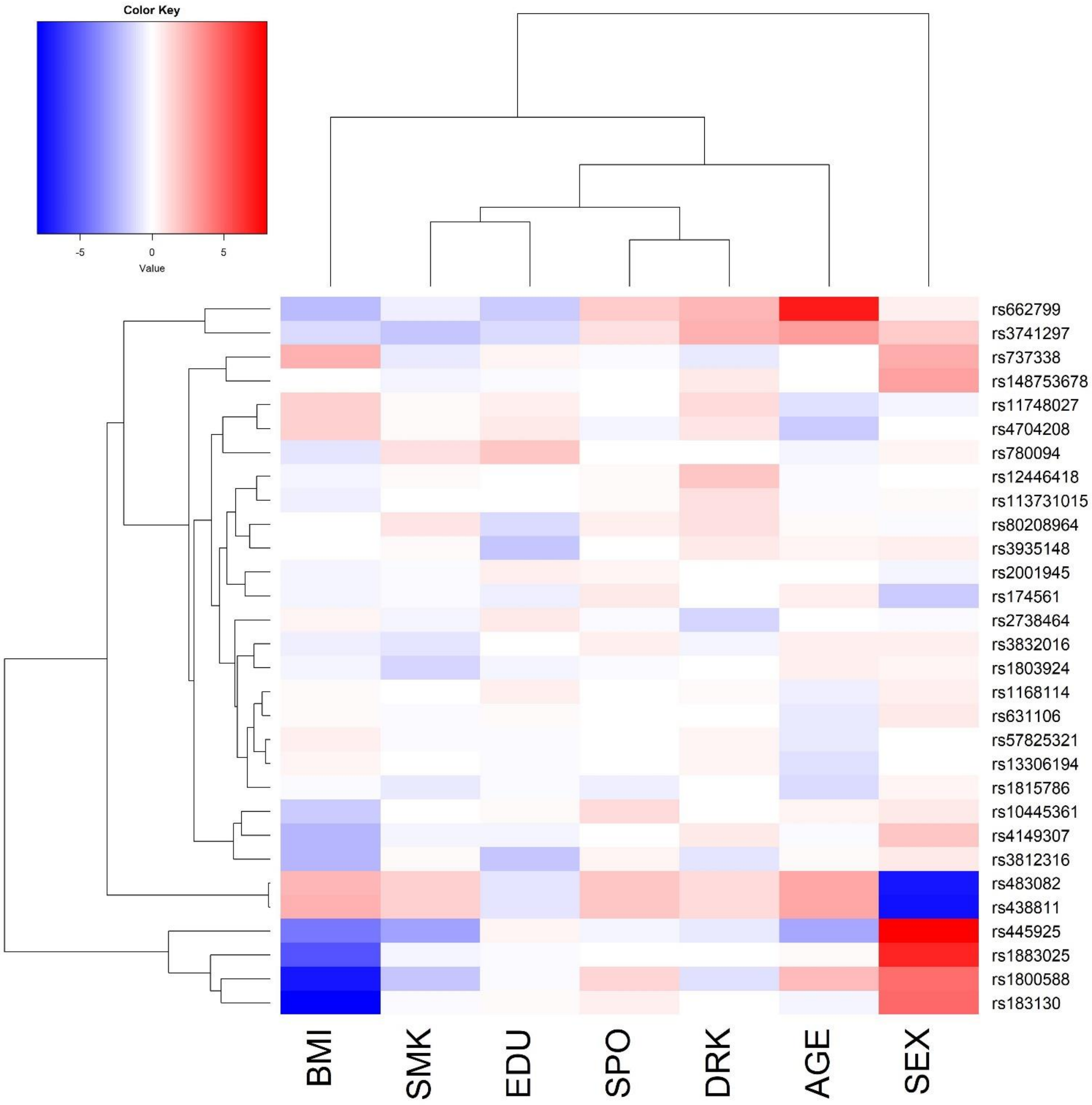

Figure S17. The phylogenetic heat map of the gene-environment interaction analysis for high-density lipoprotein cholesterol (HDL)

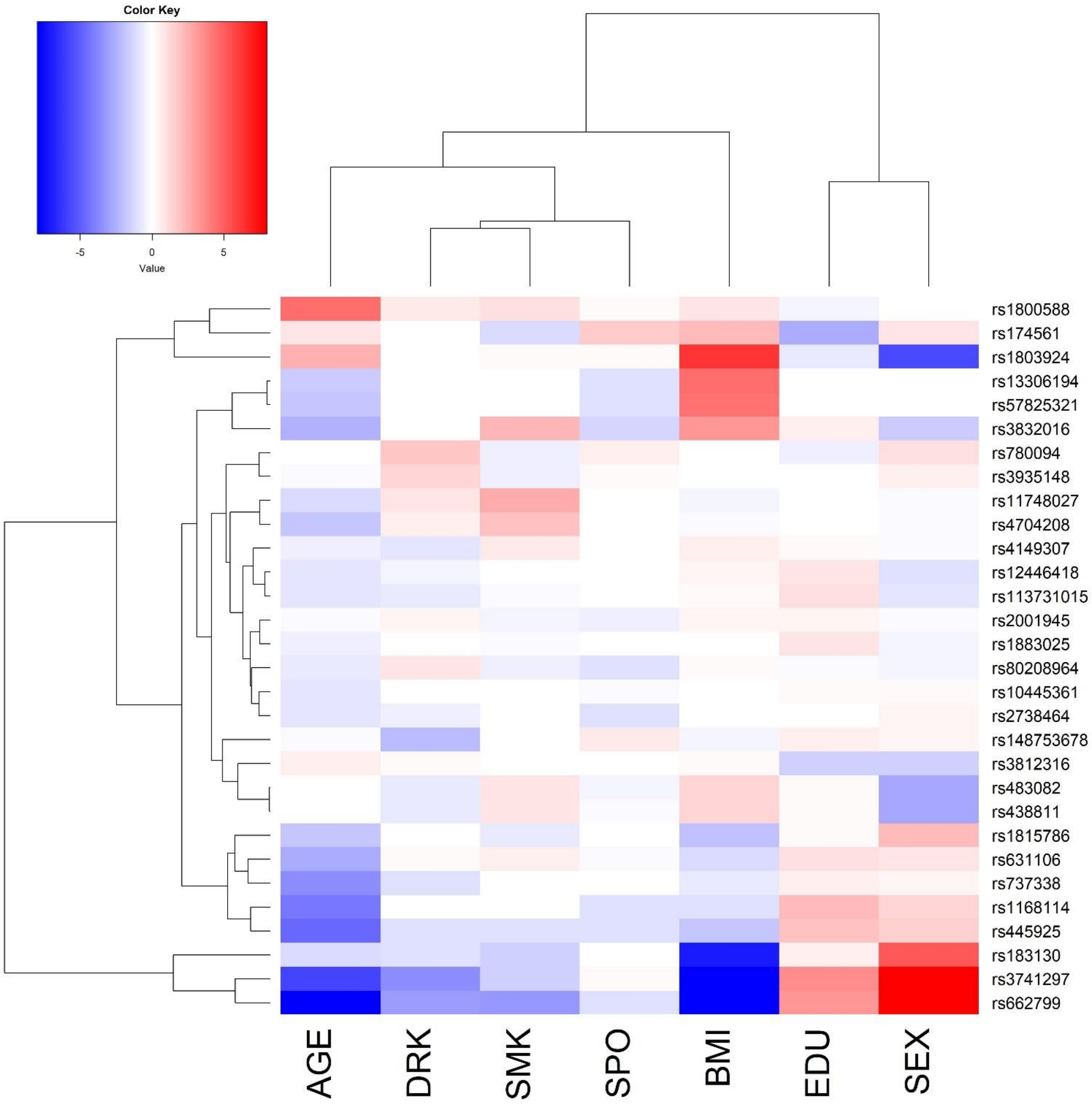

Figure S18. The phylogenetic heat map of the gene-environment interaction analysis for low-density lipoprotein cholesterol (LDL)

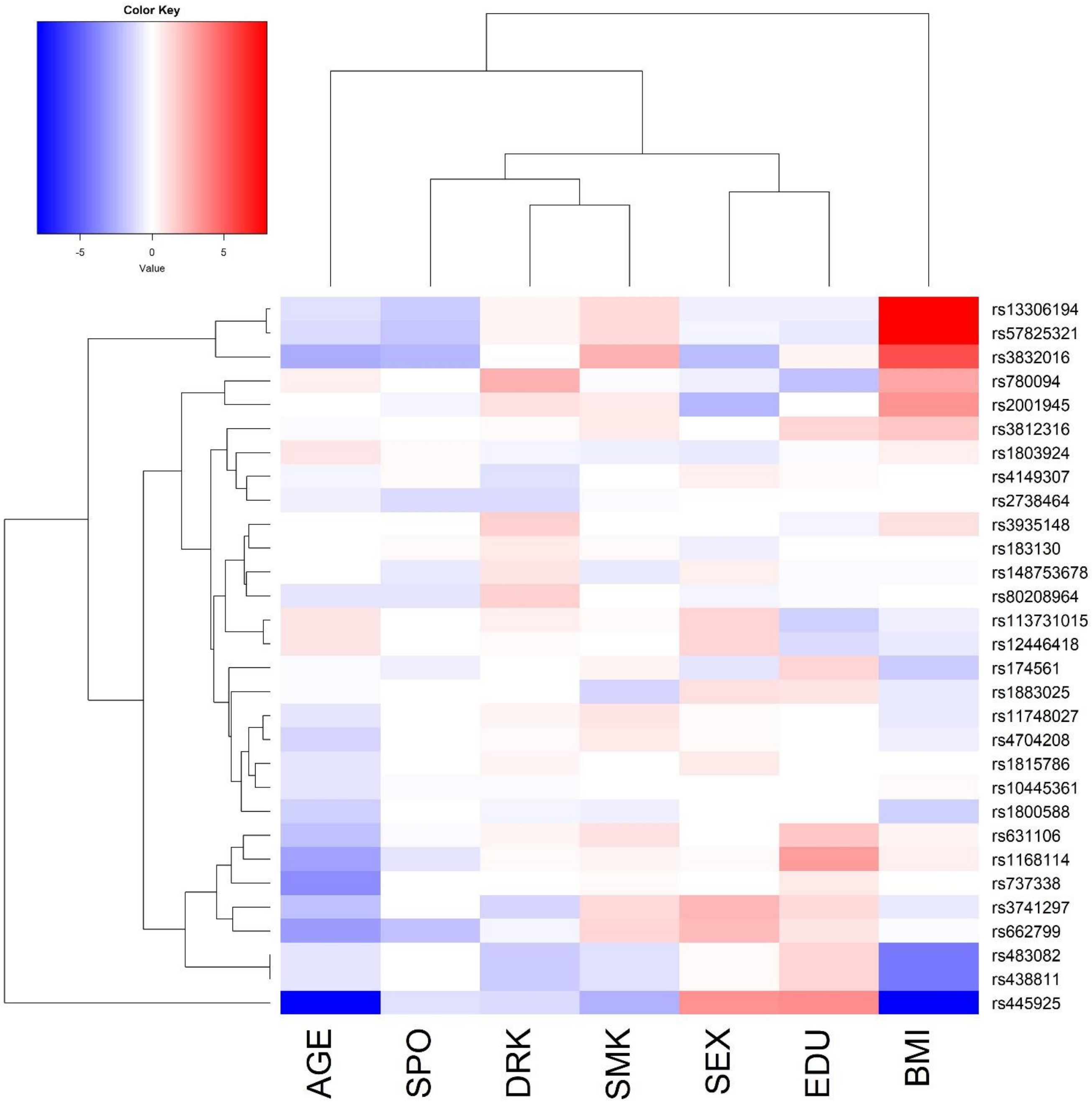

Figure S19. The phylogenetic heat map of the gene-environment interaction analysis for total cholesterol (TCHO)

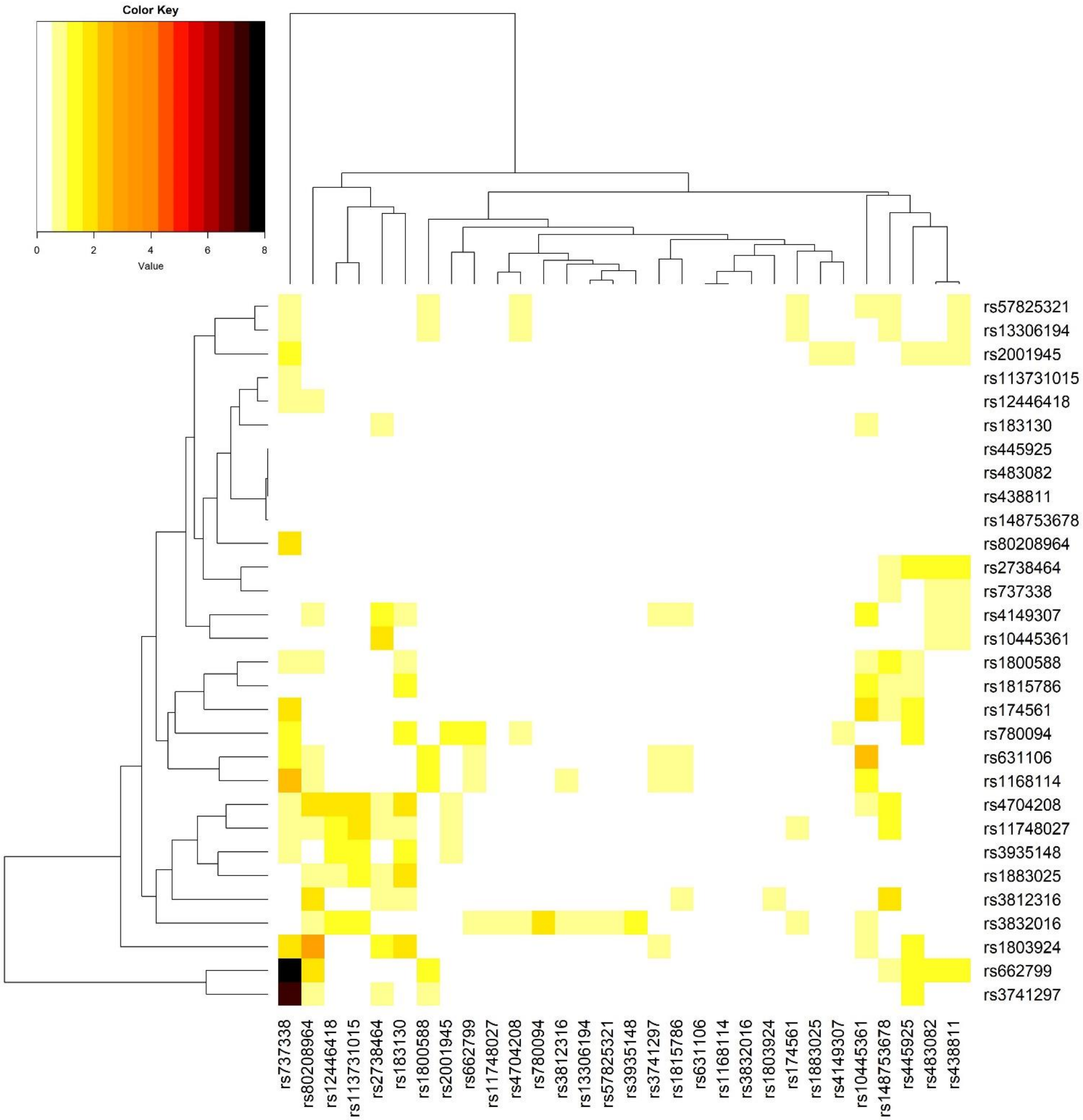

Figure S20. The phylogenetic heat map of the gene-gene interaction analysis for high-density lipoprotein cholesterol (HDL)

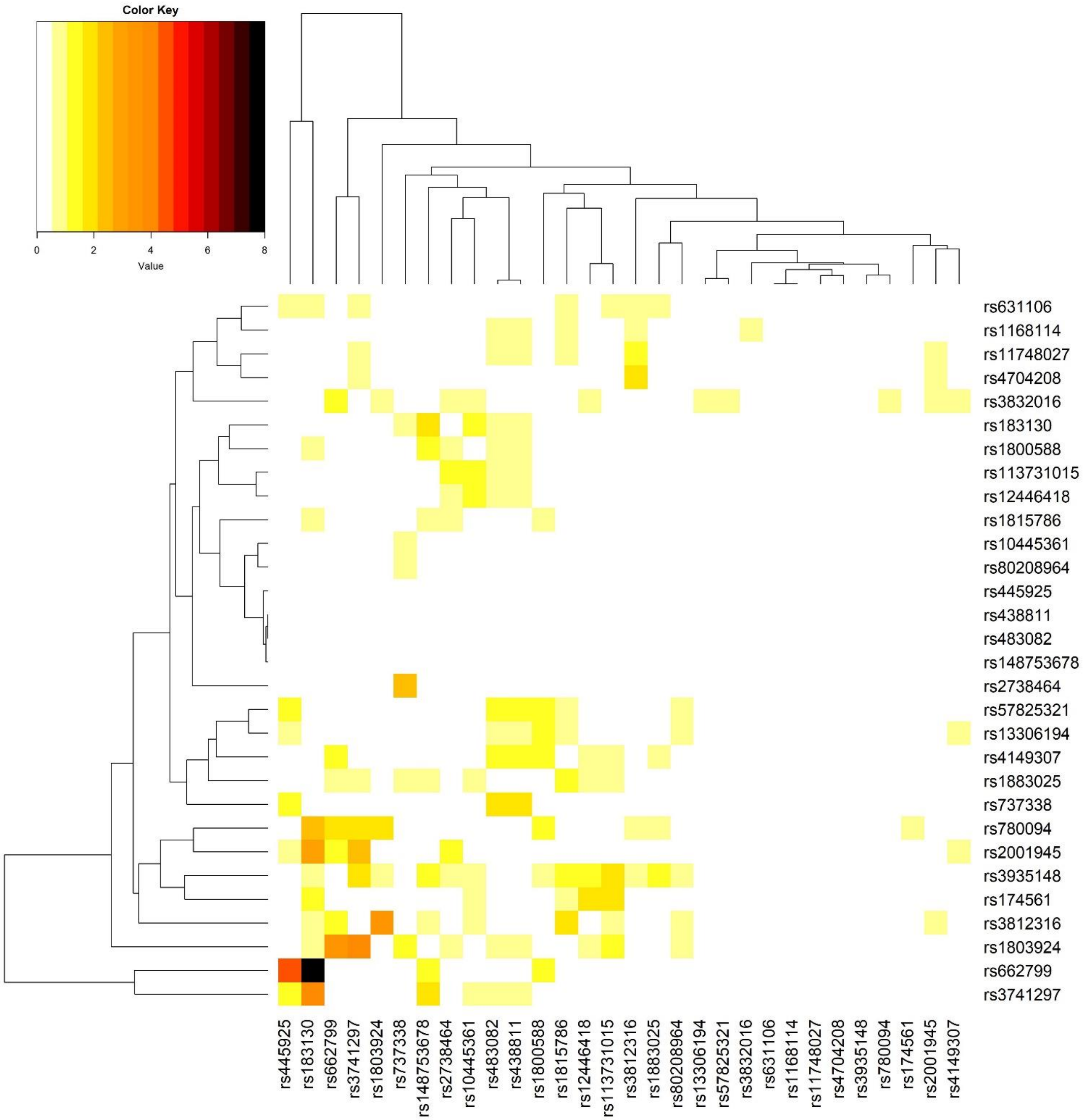

Figure S21. The phylogenetic heat map of the gene-gene interaction analysis for low-density lipoprotein cholesterol (LDL)

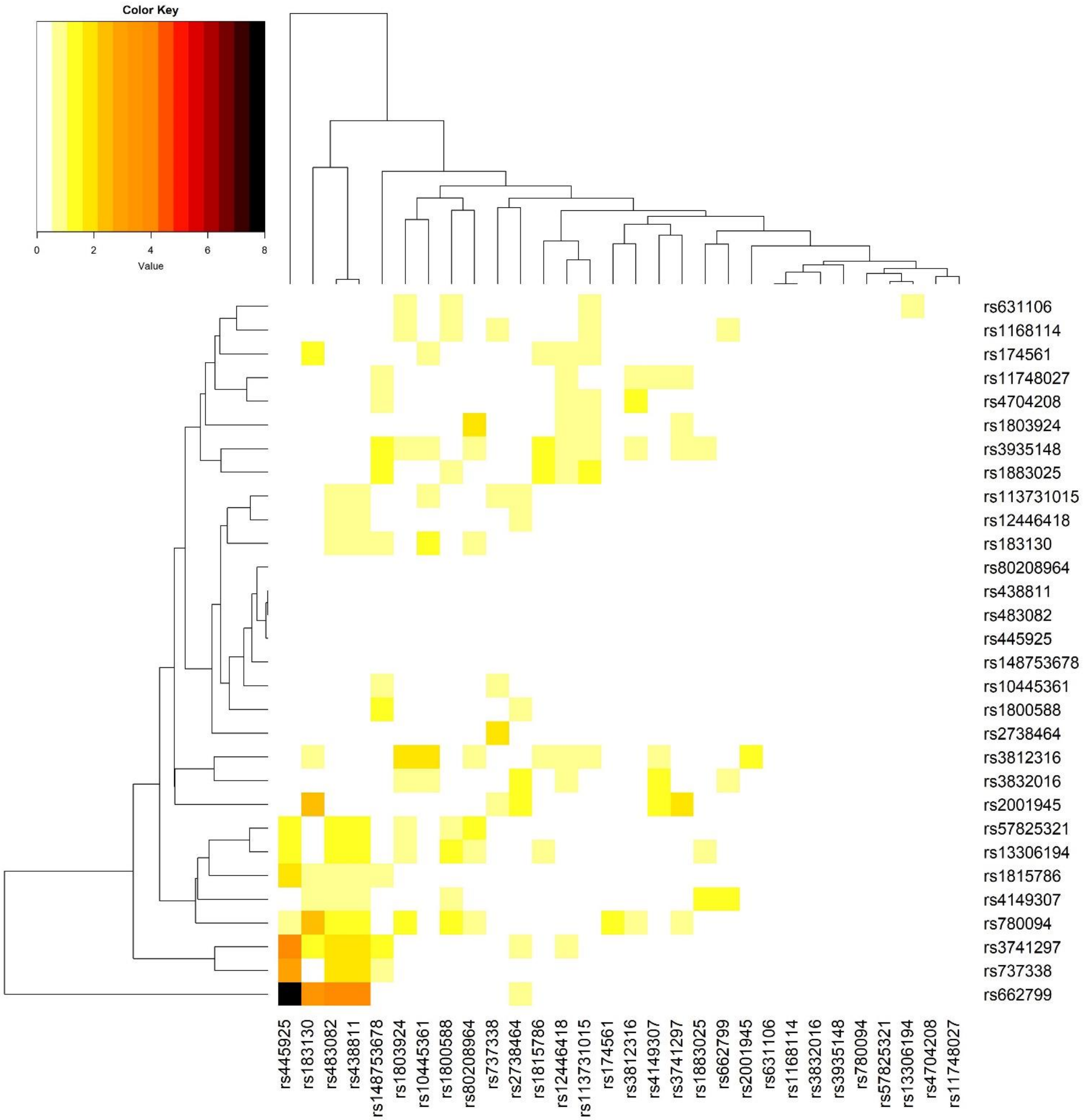

Figure S22. The phylogenetic heat map of the gene-gene interaction analysis for total cholesterol (TCHO)
